# Supplementary material for: A robust and interpretable machine learning approach using multimodal biological data to predict future pathological tau accumulation
Source: Nat Commun. 2022 Apr 7;13:1887. doi: 10.1038/s41467-022-28795-7 (PMC8989879; doi:10.1038/s41467-022-28795-7)
Supplement: Supplementary file 1 — Supplementary Information [file 41467_2022_28795_MOESM1_ESM.docx]

Supplementary Materials:

A robust and interpretable machine learning approach using multimodal biological data to predict future pathological tau accumulation

**Authors:** Joseph Giorgio, William J, Jagust, Suzanne Baker, Susan M. Landau, Peter Tino, Zoe Kourtzi, for the Alzheimer’s Disease Neuroimaging Initiative

Supplementary Figure 1: Distributions of biological predictors for Clinically Stable, Clinically Declining and Alzheimer’s Clinical Syndrome groups.

Supplementary Figure 2. Effect of MRI field strength on scalar projection (BACS)

Supplementary Figure 3. ADNI 3 difference in baseline tau burden Clinically Declining vs. Clinically Stable

Supplementary Figure 4. BACS difference in baseline tau burden Clinically Declining vs. Clinically Stable

Supplementary Figure 5. Regional future annualised rate of tau accumulation CN vs. MCI

Supplementary Figure 6. Regional future annualised rate of tau accumulation across the 36 Desikan Killiany ROIs. Classification of Clinically Declining vs Clinically Stable individuals using cognitive data from the ADNI 3 sample

Supplementary Figure 7. Relationship of medial temporal lobe (MTL) grey matter density score and total intracranial volume (TIV).

Supplementary Table 1. ADNI 3 difference in baseline tau burden Clinically Declining vs. Clinically Stable.

Supplementary Table 2. Regional future annualised rate of tau accumulation Clinically Declining vs. Clinically Stable

Supplementary Table 3. Regional future annualised rate of tau accumulation CN vs. MCI

Supplementary Table 4. Regional Future Annualised Rate of Tau Accumulation Clinically Declining vs. β-amyloid Positive

Supplementary Table 5. Fitting individual variability in regional future annualised rate of tau accumulation

Supplementary Results.

Supplementary Methods Generalised Matrix Learning Vector Quantisation

**Supplementary Figures**

**
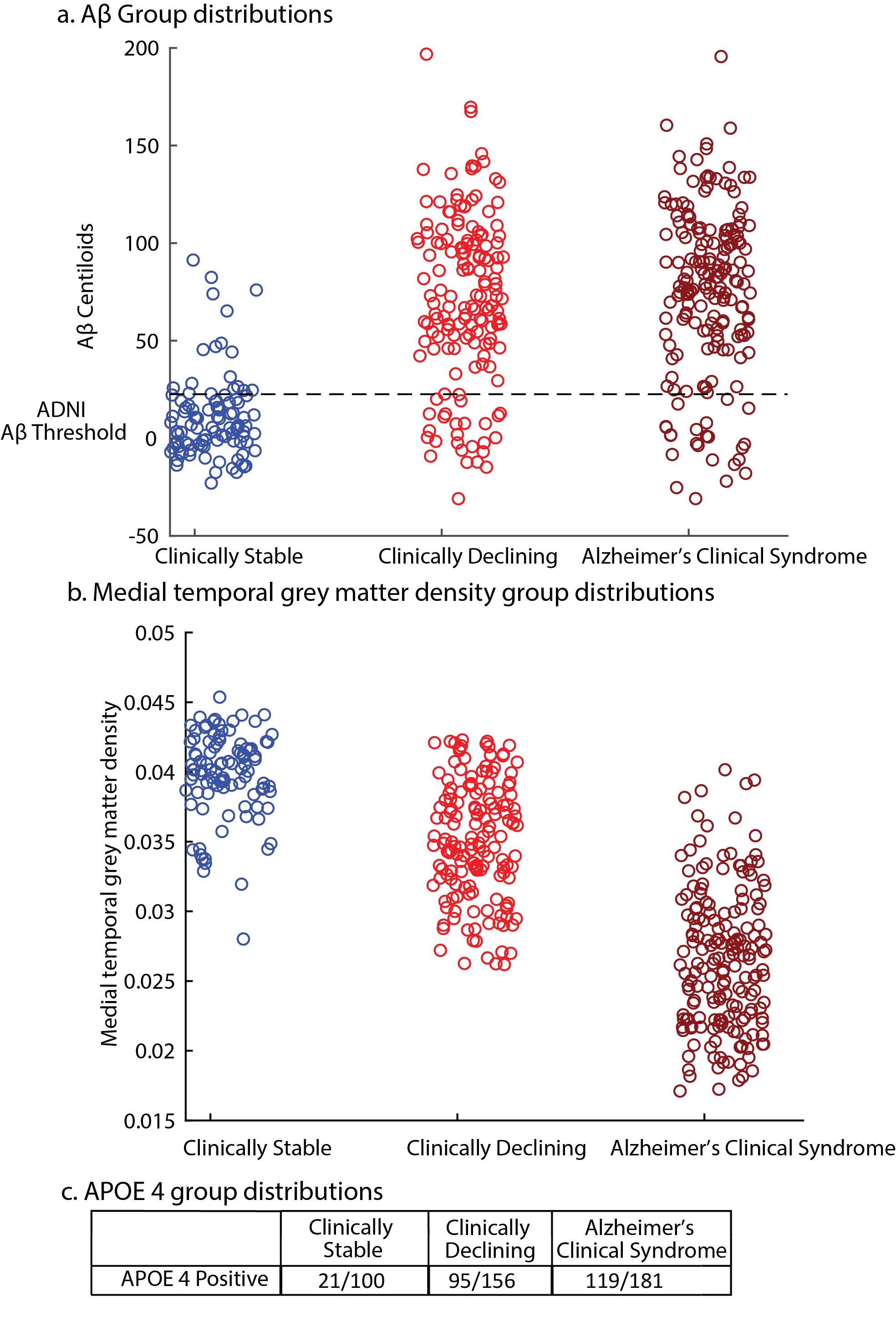
**

**Supplementary Figure 1: Distributions of biological predictors for Clinically Stable, Clinically Declining and Alzheimer’s Clinical Syndrome groups.** Distribution of the biological predictors used in the GMLVQ-scalar projection model for the ADNI2/GO sample. Blue dots indicate individuals in the Clinically Stable group, red dots indicate individuals in the Clinically Declining group and maroon dots indicate individuals in the Alzheimer’s Clinical Syndrome group. **a.** distribution of FBP (Aβ) centiloid values, the dashed horizontal line indicates the ADNI threshold of Aβ positivity (SUVR=1.11). Aβ is significantly different between Clinically Stable and Clinically Declining groups t(254)=13.2; p<0.0001 (two-tailed, two sample t-test) **b.** distribution of medial temporal grey matter density shows significant differences between Clinically Stable and Clinically Declining groups t(254)=-9.7; p<0.0001(two-tailed, two sample t-test). Further, the Clinically Declining group has greater medial temporal grey matter density than the Alzheimer’s Clinical Syndrome group t(335)=-16.74, p<0.0001(two-tailed, two sample t-test), suggesting that the Clinically Declining group is at an earlier pathophysiological stage than the Alzheimer’s Clinical Syndrome group. **c** proportions of APOE 4 positive individuals in the Clinically Stable, Clinically Declining and Alzheimer’s Clinical Syndrome groups. The Clinically Declining group has a higher proportion of APOE 4 positivity that the Clinically stable group χ2(1,254)=39.14, p<0.0001 (two-tailed, Chi square test). Source data are provided as a Source Data file.

**
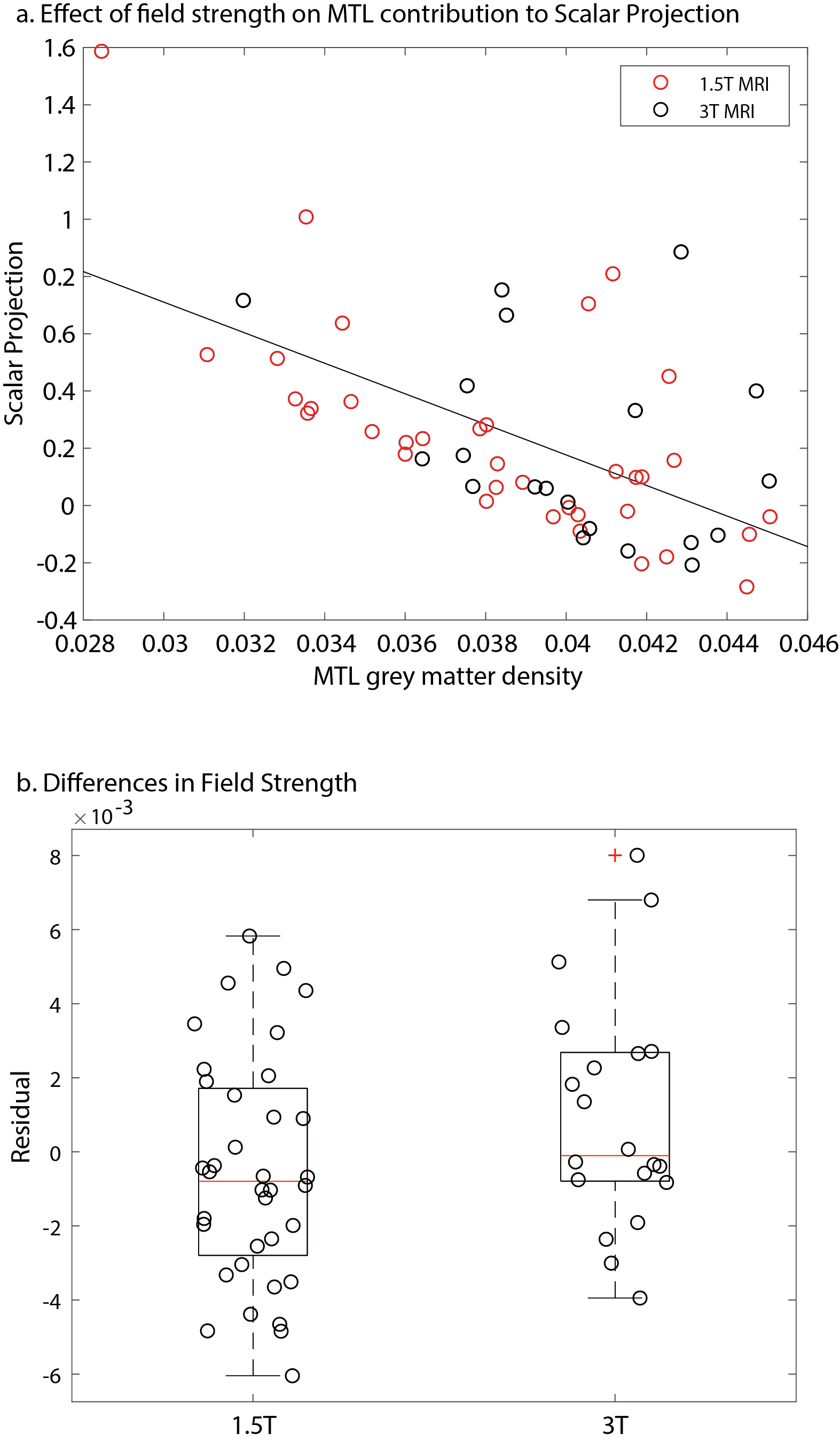
**

**Supplementary Figure 2. Effect of MRI field strength on scalar projection (BACS) a.** contribution of the medial temporal lobe (MTL) grey matter density score to the multimodal scalar projection. The black line indicates the linear best fit of these two variables. Black dots represent individuals who are scanned using 3T MRI and red dots are individuals who are scanned using 1.5T MRI. **b.** Shows the residual of the fit of the MTL grey matter density score and the scalar projection (**a.**) for individuals scanned on 1.5T (n=36) vs 3T MRI (n=20). The redline is the median of the fit, the solid black box represents the 25^th^ to 75^th^ percentile and the dashed black lines represents the range of the data. Source data are provided as a Source Data file.


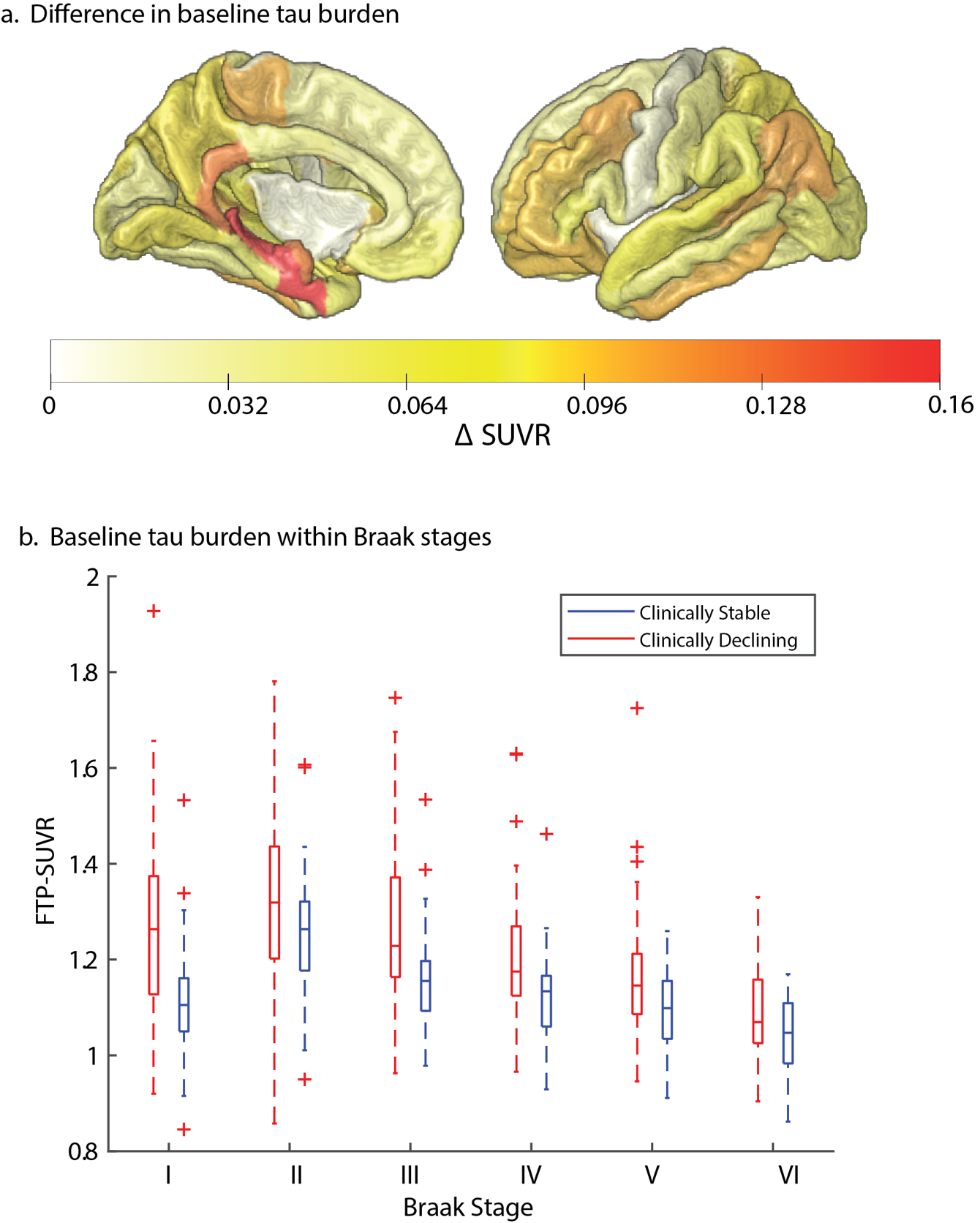
**Supplementary Figure 3. ADNI 3 difference in baseline tau burden Clinically Declining vs. Clinically Stable.** a. mean difference in baseline tau burden across the 36 Desikan-Killiany in the ADNI 3 sample. b. group differences in baseline tau burden across the six Braak stages, blue boxes show the distribution of baseline SUVR for the Clinically Stable group (n=100), red boxes show the distribution of baseline SUVR for the Clinically Declining group (n=156). The middle line is the median of the fit, the solid boxes represents the 25th to 75th percentile and the dashed lines represent the range of the data, red crosses are outliers from the distribution. Source data are provided as a Source Data file.


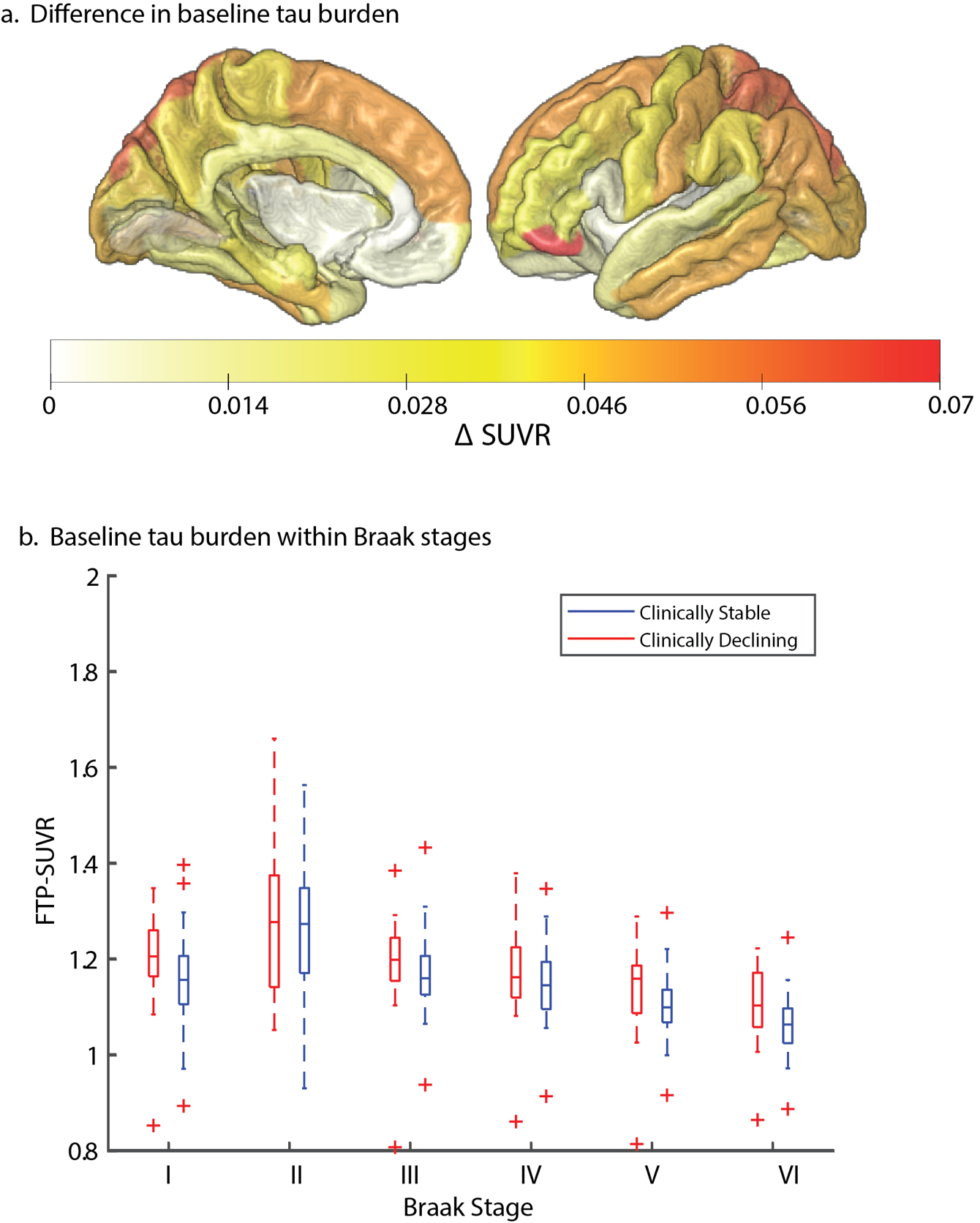

**Supplementary Figure 4. BACS difference in baseline tau burden Clinically Declining vs. Clinically Stable.** a. mean difference in baseline tau burden across the 36 Desikan-Killiany in the BACS sample. b. group differences in baseline tau burden across the six Braak stages, blue boxes show the distribution of baseline SUVR for the Clinically Stable group (n=39), red boxes show the distribution of baseline SUVR for the Clinically Declining group (n=17). The middle line is the median of the fit, the solid boxes represents the 25th to 75th percentile and the dashed lines represent the range of the data, red crosses are outliers from the distribution. Source data are provided as a Source Data file.


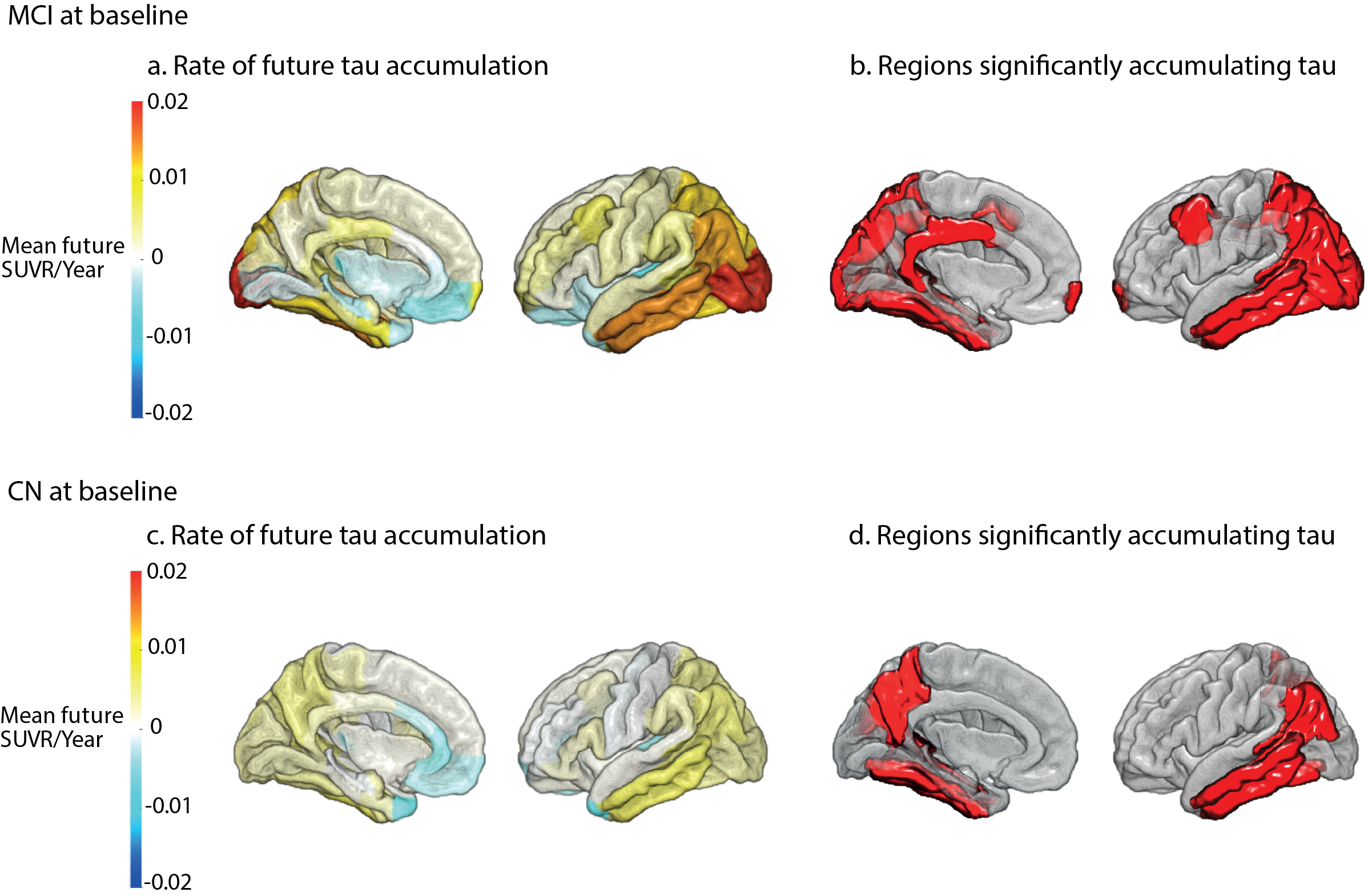


**Supplementary Figure 5. Regional future annualised rate of tau accumulation. CN vs MCI individuals from the ADNI 3 sample across the 36 Desikan Killiany ROIs. a.** Mean future annualised rate of tau accumulation for MCI. **b.** The regions in red are significantly predicted to accumulate tau for MCI individuals (one-sided t-test (right) against 0 at p<0.05 uncorrected). **c.** Mean future annualised rate of tau accumulation for Cognitively Normal (CN). **d.** The regions in red are significantly predicted to accumulate tau for Cognitively Normal individuals (one-sided t-test (right) against 0 at p<0.05 uncorrected). Source data are provided as a Source Data file.


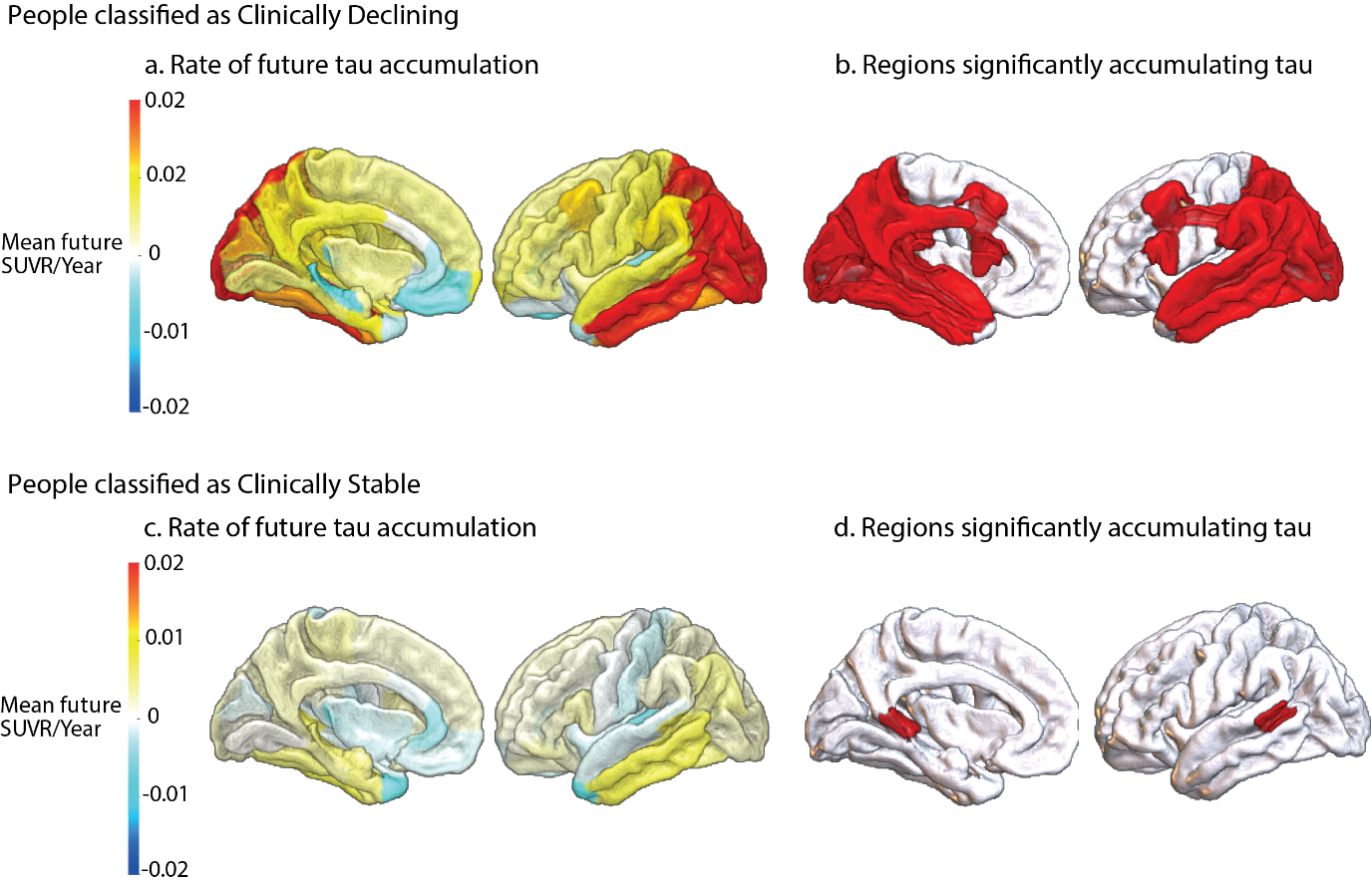


**Supplementary Figure 6. Regional future annualised rate of tau accumulation across the 36 Desikan Killiany ROIs. Classification of Clinically Declining vs Clinically Stable individuals using cognitive data from the ADNI 3 sample. a.** Mean future annualised rate of tau accumulation for Clinically Declining. **b.** The regions in red are significantly predicted to accumulate tau for Clinically Declining individuals (one-sided t-test (right) against 0 at p<0.05 uncorrected). **c.** Mean future annualised rate of tau accumulation for Clinically Stable (CN). **d.** The regions in red are significantly predicted to accumulate tau for Clinically Stable individuals (one-sided t-test (right) against 0 at p<0.05 uncorrected).

**
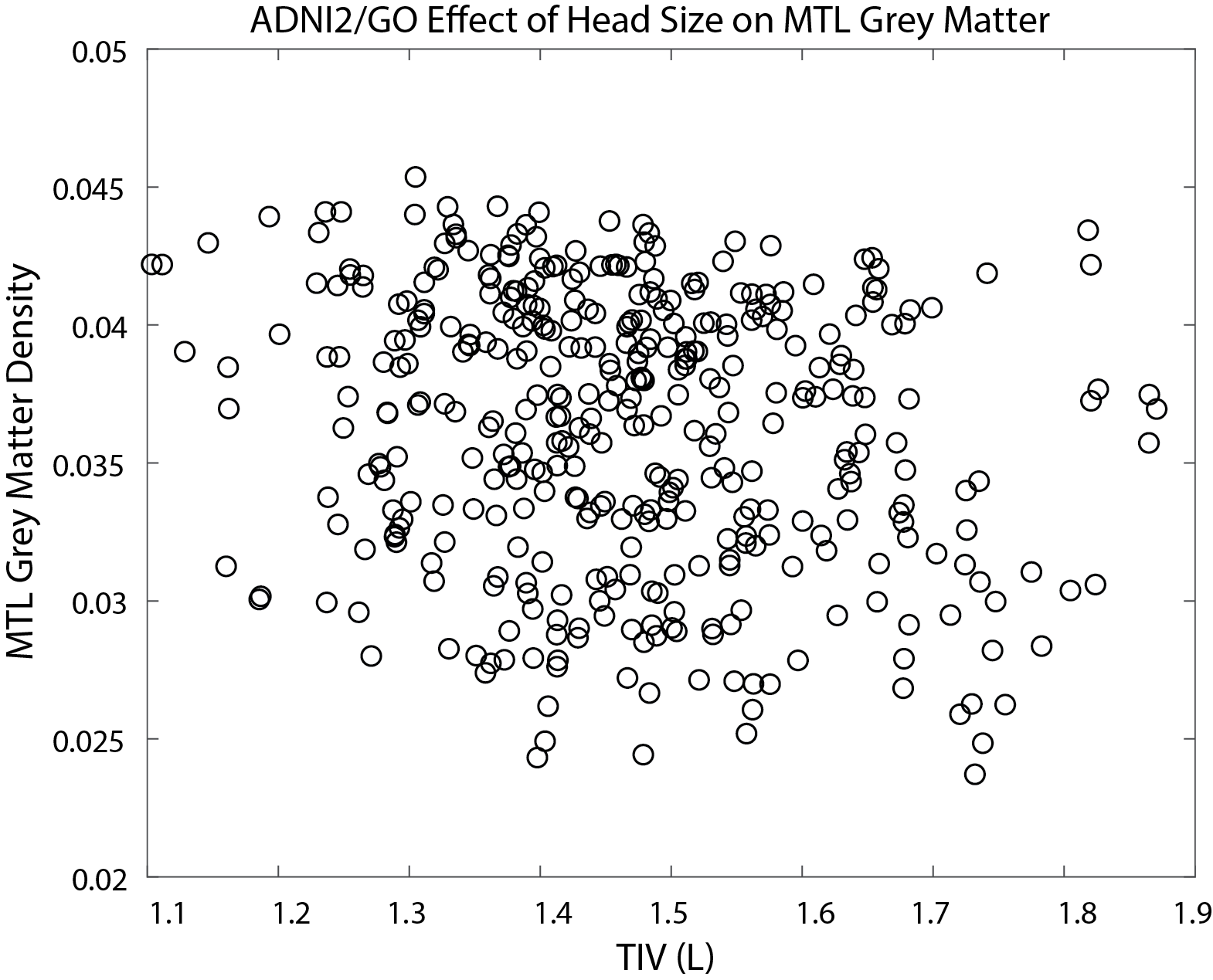
**

**Supplementary Figure 7. Relationship of medial temporal lobe (MTL) grey matter density score and total intracranial volume (TIV).** The relationship between the MTL grey matter density values and total intracranial volume for the ADNI2/GO training sample. The shared variance between these two variables is R^2^=3.7%. Source data are provided as a Source Data file.

**Supplementary Tables**

| Braak Stage |  | Mean Clinically Stable | Mean Clinically Declining | t-stat ROI | t-stat Braak | p-val ROI | p-val Braak |
| --- | --- | --- | --- | --- | --- | --- | --- |
| 1 | ENTORHINAL | 1.108 | 1.275 | 5.596 | 5.596 | <0.001 | <0.001 |
| 2 | HIPPOCAMPUS | 1.251 | 1.325 | 2.568 | 2.568 | 0.012 | 0.012 |
| 3 | PARAHIPPOCAMPAL | 1.111 | 1.214 | 4.366 | 4.773 | <0.001 | <0.001 |
|  | FUSIFORM | 1.193 | 1.308 | 4.185 |  | <0.001 |  |
|  | LINGUAL | 1.095 | 1.152 | 3.219 |  | 0.002 |  |
|  | AMYGDALA | 1.214 | 1.397 | 5.058 |  | <0.001 |  |
| 4 | MIDDLETEMPORAL | 1.168 | 1.278 | 3.576 | 3.81 | 0.001 | <0.001 |
|  | CAUDALANTERIORCINGULATE | 1.058 | 1.098 | 2.026 |  | 0.045 |  |
|  | ROSTRALANTERIORCINGULATE | 1.084 | 1.125 | 1.961 |  | 0.052 |  |
|  | POSTERIORCINGULATE | 1.098 | 1.187 | 3.723 |  | <0.001 |  |
|  | ISTHMUSCINGULATE | 1.095 | 1.196 | 3.820 |  | <0.001 |  |
|  | INSULA | 1.116 | 1.178 | 3.039 |  | 0.003 |  |
|  | INFERIORTEMPORAL | 1.205 | 1.335 | 3.890 |  | <0.001 |  |
|  | TEMPORALPOLE | 1.103 | 1.195 | 3.593 |  | <0.001 |  |
| 5 | SUPERIORFRONTAL | 0.995 | 1.077 | 3.707 | 3.469 | <0.001 | <0.001 |
|  | LATERALORBITOFRONTAL | 1.194 | 1.258 | 2.964 |  | 0.004 |  |
|  | MEDIALORBITOFRONTAL | 1.121 | 1.185 | 3.118 |  | 0.002 |  |
|  | FRONTALPOLE | 0.973 | 1.022 | 2.061 |  | 0.042 |  |
|  | CAUDALMIDDLEFRONTAL | 1.031 | 1.143 | 3.628 |  | <0.001 |  |
|  | ROSTRALMIDDLEFRONTAL | 1.049 | 1.134 | 3.107 |  | 0.002 |  |
|  | PARSOPERCULARIS | 1.087 | 1.159 | 2.786 |  | 0.006 |  |
|  | PARSORBITALIS | 1.144 | 1.186 | 1.884 |  | 0.062 |  |
|  | PARSTRIANGULARIS | 1.115 | 1.164 | 2.190 |  | 0.031 |  |
|  | LATERALOCCIPITAL | 1.095 | 1.181 | 2.730 |  | 0.007 |  |
|  | SUPRAMARGINAL | 1.087 | 1.149 | 2.747 |  | 0.007 |  |
|  | INFERIORPARIETAL | 1.130 | 1.237 | 3.475 |  | 0.001 |  |
|  | SUPERIORTEMPORAL | 1.087 | 1.149 | 2.931 |  | 0.004 |  |
|  | SUPERIORPARIETAL | 1.028 | 1.097 | 3.227 |  | 0.002 |  |
|  | PRECUNEUS | 1.112 | 1.211 | 3.976 |  | <0.001 |  |
|  | BANKSSTS | 1.211 | 1.316 | 3.193 |  | 0.002 |  |
|  | TRANSVERSETEMPORAL | 1.035 | 1.048 | 0.725 |  | 0.470 |  |
| 6 | PERICALCARINE | 1.119 | 1.168 | 2.666 | 2.534 | 0.009 | 0.012 |
|  | POSTCENTRAL | 0.979 | 0.998 | 1.179 |  | 0.241 |  |
|  | CUNEUS | 1.102 | 1.146 | 2.216 |  | 0.029 |  |
|  | PRECENTRAL | 1.001 | 1.040 | 2.171 |  | 0.032 |  |
|  | PARACENTRAL | 1.022 | 1.074 | 2.776 |  | 0.006 |  |

**Supplementary Table 1. ADNI 3 difference in baseline tau burden Clinically Declining vs. Clinically Stable.** Measures of baseline regional tau SUVR the Desikan Killiany atlas for ADNI 3 individuals grouped in the 6 Braak stages. The average tau burden and statistics describing whether a region has significantly greater tau for individuals classified as Clinically Declining (two-tailed, two sample t-test uncorrected). Source data are provided as a Source Data file.

| Braak Stage | Region | Clinically Stable (n=59) | | | Clinically Declining (n=56) | | |
| --- | --- | --- | --- | --- | --- | --- | --- |
|  |  |  | Significantly Accumulating Tau | |  | Significantly Accumulating Tau | |
|  |  | mean accumulation (SUVR/Year) | t-stat | p-val | mean accumulation (SUVR/Year) | t-stat | p-val |
| 1 | ENTORHINAL | 0.0049 | 1.177 | 0.122 | 0.0071 | 1.264 | 0.106 |
| 2 | HIPPOCAMPUS | 0.0046 | 1.312 | 0.097 | -0.0049 | -1.133 | 0.869 |
| 3 | PARAHIPPOCAMPAL | 0.0023 | 0.621 | 0.268 | 0.0047 | 1.092 | 0.140 |
|  | FUSIFORM | 0.0028 | 0.793 | 0.216 | 0.0136 | 3.044 | 0.002 |
|  | LINGUAL | 0.0008 | 0.224 | 0.412 | 0.0043 | 1.159 | 0.126 |
|  | AMYGDALA | 0.0033 | 0.897 | 0.187 | 0.0059 | 1.227 | 0.113 |
| 4 | MIDDLETEMPORAL | 0.0050 | 1.215 | 0.115 | 0.0178 | 3.881 | 0.000 |
|  | CAUDALANTERIORCINGULATE | 0.0035 | 1.149 | 0.128 | -0.0044 | -1.519 | 0.933 |
|  | ROSTRALANTERIORCINGULATE | 0.0018 | 0.555 | 0.290 | -0.0077 | -3.027 | 0.998 |
|  | POSTERIORCINGULATE | 0.0032 | 1.201 | 0.117 | 0.0047 | 1.288 | 0.102 |
|  | ISTHMUSCINGULATE | 0.0014 | 0.511 | 0.306 | 0.0077 | 2.199 | 0.016 |
|  | INSULA | 0.0001 | 0.037 | 0.485 | 0.0017 | 0.580 | 0.282 |
|  | INFERIORTEMPORAL | 0.0051 | 1.284 | 0.102 | 0.0175 | 3.633 | 0.000 |
|  | TEMPORALPOLE | -0.0038 | -0.718 | 0.762 | -0.0041 | -0.955 | 0.828 |
| 5 | SUPERIORFRONTAL | 0.0029 | 0.912 | 0.183 | 0.0012 | 0.486 | 0.314 |
|  | LATERALORBITOFRONTAL | 0.0029 | 0.916 | 0.182 | -0.0018 | -0.684 | 0.752 |
|  | MEDIALORBITOFRONTAL | 0.0010 | 0.305 | 0.381 | -0.0062 | -2.069 | 0.978 |
|  | FRONTALPOLE | 0.0049 | 1.109 | 0.136 | -0.0002 | -0.040 | 0.516 |
|  | CAUDALMIDDLEFRONTAL | 0.0043 | 1.430 | 0.079 | 0.0057 | 1.628 | 0.055 |
|  | ROSTRALMIDDLEFRONTAL | 0.0032 | 0.900 | 0.186 | 0.0016 | 0.513 | 0.305 |
|  | PARSOPERCULARIS | 0.0032 | 0.977 | 0.166 | 0.0036 | 1.541 | 0.065 |
|  | PARSORBITALIS | 0.0050 | 0.962 | 0.170 | 0.0000 | 0.003 | 0.499 |
|  | PARSTRIANGULARIS | 0.0028 | 0.675 | 0.251 | 0.0027 | 0.892 | 0.188 |
|  | LATERALOCCIPITAL | 0.0025 | 0.551 | 0.292 | 0.0196 | 3.288 | 0.001 |
|  | SUPRAMARGINAL | -0.0001 | -0.029 | 0.511 | 0.0100 | 2.703 | 0.005 |
|  | INFERIORPARIETAL | 0.0013 | 0.358 | 0.361 | 0.0190 | 4.122 | 0.000 |
|  | SUPERIORTEMPORAL | 0.0011 | 0.327 | 0.372 | 0.0037 | 1.103 | 0.137 |
|  | SUPERIORPARIETAL | 0.0015 | 0.378 | 0.354 | 0.0153 | 3.421 | 0.001 |
|  | PRECUNEUS | 0.0008 | 0.312 | 0.378 | 0.0094 | 2.785 | 0.004 |
|  | BANKSSTS | 0.0016 | 0.423 | 0.337 | 0.0130 | 3.245 | 0.001 |
|  | TRANSVERSETEMPORAL | -0.0031 | -1.000 | 0.839 | -0.0046 | -1.223 | 0.887 |
| 6 | PERICALCARINE | 0.0020 | 0.619 | 0.269 | 0.0027 | 0.707 | 0.241 |
|  | POSTCENTRAL | 0.0010 | 0.279 | 0.391 | 0.0032 | 0.985 | 0.164 |
|  | CUNEUS | -0.0003 | -0.086 | 0.534 | 0.0089 | 2.149 | 0.018 |
|  | PRECENTRAL | 0.0017 | 0.549 | 0.292 | 0.0025 | 0.936 | 0.177 |
|  | PARACENTRAL | 0.0026 | 0.810 | 0.210 | 0.0050 | 1.469 | 0.074 |
|  | Mean | 0.0022 |  |  | 0.0050 |  |  |

**Supplementary Table 2. Regional future annualised rate of tau accumulation** Measures of future regional annualised rate of tau accumulation taken from the Desikan Killiany atlas for ADNI 3 individuals within the 6 Braak stages. The mean future annualised rate of tau accumulation and test statistics describing whether a region significantly accumulates tau for individuals classified as Clinically Stable (Left block) and for individuals classified as Clinically Declining (Right block) (one-sided t-test (right) against 0, uncorrected). Source data are provided as a Source Data file.

| Braak Stage | Region | Cognitively Normal (n=72) | | | MCI (n=43) | | |
| --- | --- | --- | --- | --- | --- | --- | --- |
|  |  |  | Significantly Accumulating Tau | |  | Significantly Accumulating Tau | |
|  |  | mean accumulation (SUVR/Year) | t-stat | p-val | mean accumulation (SUVR/Year) | t-stat | p-val |
| 1 | ENTORHINAL | 0.004 | 0.984 | 0.164 | 0.010 | 1.442 | 0.078 |
| 2 | HIPPOCAMPUS | 0.001 | 0.253 | 0.401 | -0.002 | -0.312 | 0.622 |
| 3 | PARAHIPPOCAMPAL | 0.002 | 0.547 | 0.293 | 0.006 | 1.311 | 0.098 |
|  | FUSIFORM | 0.005 | 1.844 | 0.035 | 0.013 | 2.142 | 0.019 |
|  | LINGUAL | 0.004 | 1.212 | 0.115 | 0.000 | 0.045 | 0.482 |
|  | AMYGDALA | 0.005 | 1.226 | 0.112 | 0.005 | 0.898 | 0.187 |
| 4 | MIDDLETEMPORAL | 0.008 | 2.230 | 0.014 | 0.016 | 2.915 | 0.003 |
|  | CAUDALANTERIORCINGULATE | -0.001 | -0.456 | 0.675 | 0.001 | 0.385 | 0.351 |
|  | ROSTRALANTERIORCINGULATE | -0.004 | -1.745 | 0.957 | -0.001 | -0.152 | 0.560 |
|  | POSTERIORCINGULATE | 0.003 | 0.815 | 0.209 | 0.006 | 2.085 | 0.022 |
|  | ISTHMUSCINGULATE | 0.004 | 1.316 | 0.096 | 0.005 | 1.873 | 0.034 |
|  | INSULA | 0.002 | 0.754 | 0.227 | -0.001 | -0.143 | 0.556 |
|  | INFERIORTEMPORAL | 0.008 | 2.353 | 0.011 | 0.016 | 2.651 | 0.006 |
|  | TEMPORALPOLE | -0.005 | -1.290 | 0.899 | -0.002 | -0.284 | 0.611 |
| 5 | SUPERIORFRONTAL | 0.002 | 0.704 | 0.242 | 0.003 | 0.753 | 0.228 |
|  | LATERALORBITOFRONTAL | 0.002 | 0.773 | 0.221 | -0.002 | -0.412 | 0.659 |
|  | MEDIALORBITOFRONTAL | -0.002 | -0.575 | 0.716 | -0.004 | -1.028 | 0.845 |
|  | FRONTALPOLE | -0.002 | -0.628 | 0.734 | 0.011 | 1.721 | 0.046 |
|  | CAUDALMIDDLEFRONTAL | 0.003 | 1.043 | 0.150 | 0.008 | 2.204 | 0.017 |
|  | ROSTRALMIDDLEFRONTAL | 0.001 | 0.332 | 0.370 | 0.005 | 1.091 | 0.141 |
|  | PARSOPERCULARIS | 0.004 | 1.566 | 0.061 | 0.003 | 0.736 | 0.233 |
|  | PARSORBITALIS | 0.003 | 0.638 | 0.263 | 0.002 | 0.366 | 0.358 |
|  | PARSTRIANGULARIS | 0.003 | 0.942 | 0.175 | 0.002 | 0.520 | 0.303 |
|  | LATERALOCCIPITAL | 0.005 | 1.207 | 0.116 | 0.021 | 2.783 | 0.004 |
|  | SUPRAMARGINAL | 0.004 | 1.404 | 0.082 | 0.006 | 1.438 | 0.079 |
|  | INFERIORPARIETAL | 0.007 | 2.034 | 0.023 | 0.015 | 2.645 | 0.006 |
|  | SUPERIORTEMPORAL | 0.002 | 0.509 | 0.306 | 0.004 | 0.966 | 0.170 |
|  | SUPERIORPARIETAL | 0.006 | 1.569 | 0.061 | 0.013 | 2.293 | 0.013 |
|  | PRECUNEUS | 0.006 | 2.177 | 0.016 | 0.003 | 0.897 | 0.188 |
|  | BANKSSTS | 0.007 | 1.858 | 0.034 | 0.008 | 1.828 | 0.037 |
|  | TRANSVERSETEMPORAL | -0.003 | -0.952 | 0.828 | -0.005 | -1.336 | 0.906 |
| 6 | PERICALCARINE | 0.004 | 1.436 | 0.078 | -0.001 | -0.283 | 0.611 |
|  | POSTCENTRAL | 0.001 | 0.388 | 0.350 | 0.004 | 0.862 | 0.197 |
|  | CUNEUS | 0.004 | 1.139 | 0.129 | 0.004 | 1.020 | 0.157 |
|  | PRECENTRAL | 0.001 | 0.295 | 0.385 | 0.004 | 1.227 | 0.113 |
|  | PARACENTRAL | 0.004 | 1.326 | 0.095 | 0.003 | 0.920 | 0.181 |
|  | Mean | 0.003 |  |  | 0.005 |  |  |

**Supplementary Table 3. Regional future annualised rate of tau accumulation CN vs MCI** Measures of future regional annualised rate of tau accumulation taken from the Desikan Killiany atlas for ADNI 3 individuals within the 6 Braak stages. The mean future annualised rate of tau accumulation and test statistics describing whether a region significantly accumulates tau for Cognitively Normal individuals and for MCI individuals (Right block) (one-sided t-test (right) against 0, uncorrected). Source data are provided as a Source Data file.

| Braak Stage | Region | Clinically Declining | | | | β-amyloid Positive | | | | Clinically Declining vs.  β-amyloid Positive |
| --- | --- | --- | --- | --- | --- | --- | --- | --- | --- | --- |
|  |  |  | Significantly Accumulating Tau | | |  | Significantly Accumulating Tau | | |  |
|  |  | mean accumulation (SUVR/Year) | t-stat | p-val | sample size | mean accumulation (SUVR/Year) | t-stat | p-val | sample size | relative difference in sample size |
| 1 | ENTORHINAL | 0.0071 | 1.264 | 0.106 | 3467 | 0.007265 | 1.385973 | 0.085443 | 3142 | 1.10 |
| 2 | HIPPOCAMPUS | -0.0049 | -1.133 | 0.869 | NaN | -0.00172 | -0.43745 | 0.668323 | NaN | NaN |
| 3 | PARAHIPPOCAMPAL | 0.0047 | 1.092 | 0.140 | 4648 | 0.003522 | 0.845455 | 0.200608 | 8442 | 0.45 |
|  | FUSIFORM | 0.0136 | 3.044 | 0.002 | 598 | 0.01253 | 2.898793 | 0.002613 | 719 | 0.16 |
|  | LINGUAL | 0.0043 | 1.159 | 0.126 | 4125 | 0.00502 | 1.374027 | 0.087272 | 3197 | 1.29 |
|  | AMYGDALA | 0.0059 | 1.227 | 0.113 | 3682 | 0.007128 | 1.629791 | 0.054193 | 2272 | 1.62 |
| 4 | MIDDLETEMPORAL | 0.0178 | 3.881 | 0.000 | 368 | 0.014402 | 3.283735 | 0.000856 | 560 | 0.34 |
|  | CAUDALANTERIORCINGULATE | -0.0044 | -1.519 | 0.933 | NaN | -0.00358 | -1.27837 | 0.896979 | NaN | NaN |
|  | ROSTRALANTERIORCINGULATE | -0.0077 | -3.027 | 0.998 | NaN | -0.00714 | -2.94979 | 0.997736 | NaN | NaN |
|  | POSTERIORCINGULATE | 0.0047 | 1.288 | 0.102 | 3340 | 0.005215 | 1.479792 | 0.07208 | 2756 | 1.21 |
|  | ISTHMUSCINGULATE | 0.0077 | 2.199 | 0.016 | 1146 | 0.00619 | 1.852004 | 0.034473 | 1760 | 0.35 |
|  | INSULA | 0.0017 | 0.580 | 0.282 | 16440 | 0.001379 | 0.505238 | 0.307621 | 23639 | 0.30 |
|  | INFERIORTEMPORAL | 0.0175 | 3.633 | 0.000 | 420 | 0.016683 | 3.728422 | 0.000215 | 435 | 0.03 |
|  | TEMPORALPOLE | -0.0041 | -0.955 | 0.828 | NaN | -0.00376 | -0.93591 | 0.823464 | NaN | NaN |
| 5 | SUPERIORFRONTAL | 0.0012 | 0.486 | 0.314 | 23459 | 0.000404 | 0.163388 | 0.435381 | 226038 | 0.90 |
|  | LATERALORBITOFRONTAL | -0.0018 | -0.684 | 0.752 | NaN | -0.00218 | -0.83356 | 0.796081 | NaN | NaN |
|  | MEDIALORBITOFRONTAL | -0.0062 | -2.069 | 0.978 | NaN | -0.00545 | -1.87369 | 0.967077 | NaN | NaN |
|  | FRONTALPOLE | -0.0002 | -0.040 | 0.516 | NaN | -0.00194 | -0.40422 | 0.656256 | NaN | NaN |
|  | CAUDALMIDDLEFRONTAL | 0.0057 | 1.628 | 0.055 | 2091 | 0.003604 | 1.141774 | 0.129042 | 4629 | 0.55 |
|  | ROSTRALMIDDLEFRONTAL | 0.0016 | 0.513 | 0.305 | 21034 | 0.000255 | 0.090245 | 0.464196 | 740916 | 0.97 |
|  | PARSOPERCULARIS | 0.0036 | 1.541 | 0.065 | 2334 | 0.0025 | 0.96517 | 0.169167 | 6478 | 0.64 |
|  | PARSORBITALIS | 0.0000 | 0.003 | 0.499 | 8.45E+08 | -0.00277 | -0.61285 | 0.728854 | NaN | NaN |
|  | PARSTRIANGULARIS | 0.0027 | 0.892 | 0.188 | 6958 | 0.000124 | 0.040111 | 0.484069 | 3750535 | 0.99 |
|  | LATERALOCCIPITAL | 0.0196 | 3.288 | 0.001 | 513 | 0.015625 | 2.887378 | 0.002697 | 724 | 0.29 |
|  | SUPRAMARGINAL | 0.0100 | 2.703 | 0.005 | 758 | 0.007123 | 1.971982 | 0.026613 | 1552 | 0.51 |
|  | INFERIORPARIETAL | 0.0190 | 4.122 | 0.000 | 326 | 0.014884 | 3.322016 | 0.000763 | 547 | 0.40 |
|  | SUPERIORTEMPORAL | 0.0037 | 1.103 | 0.137 | 4550 | 0.001488 | 0.434527 | 0.332732 | 31959 | 0.86 |
|  | SUPERIORPARIETAL | 0.0153 | 3.421 | 0.001 | 474 | 0.01178 | 2.784474 | 0.003581 | 779 | 0.39 |
|  | PRECUNEUS | 0.0094 | 2.785 | 0.004 | 715 | 0.007934 | 2.454491 | 0.008513 | 1002 | 0.29 |
|  | BANKSSTS | 0.0130 | 3.245 | 0.001 | 526 | 0.009368 | 2.172949 | 0.016872 | 1278 | 0.59 |
|  | TRANSVERSETEMPORAL | -0.0046 | -1.223 | 0.887 | NaN | -0.0036 | -0.99262 | 0.837558 | NaN | NaN |
| 6 | PERICALCARINE | 0.0027 | 0.707 | 0.241 | 11080 | 0.002568 | 0.702962 | 0.242399 | 12212 | 0.09 |
|  | POSTCENTRAL | 0.0032 | 0.985 | 0.164 | 5707 | 0.001053 | 0.326228 | 0.372694 | 56700 | 0.90 |
|  | CUNEUS | 0.0089 | 2.149 | 0.018 | 1200 | 0.005353 | 1.378229 | 0.086625 | 3177 | 0.62 |
|  | PRECENTRAL | 0.0025 | 0.936 | 0.177 | 6318 | 0.000536 | 0.209812 | 0.417263 | 137076 | 0.95 |
|  | PARACENTRAL | 0.0050 | 1.469 | 0.074 | 2566 | 0.004181 | 1.311466 | 0.097348 | 3509 | 0.27 |
|  | Mean | 0.0050 |  |  |  | 0.0037 |  |  |  |  |

**Supplementary Table 4. Regional Future Annualised Rate of Tau Accumulation Clinically Declining vs. β-amyloid Positive.** Measures of future annualised rate of tau accumulation for regions taken from the Desikan Killiany atlas within the 6 Braak stages. The mean future annualised rate of tau accumulation and test statistics describing whether a region is significantly accumulating tau for individuals classified as Clinically Declining (Left block) and for individuals who are β-amyloid positive only (Right block) (one-sided t-test (right) against 0, uncorrected). The Sample size reported is for a hypothetical clinical trial looking to observe a 25% reduction in rate of tau accumulation per region. The right most column compares the required sample size to observe the same effect for groups defined my multimodal data or amyloid status only. Source data are provided as a Source Data file.

| Braak Stage | Region | Beta Estimate (SUVR/Year) | t-stat | p-val | %Variance Explained |
| --- | --- | --- | --- | --- | --- |
| 3 | FUSIFORM | 0.028 | 3.425 | 0.001 | 21.1 |
| 4 | MIDDLETEMPORAL | 0.019 | 1.854 | 0.069 | 9.9 |
|  | ISTHMUSCINGULATE | 0.011 | 1.735 | 0.088 | 9.4 |
|  | INFERIORTEMPORAL | 0.021 | 2.009 | 0.050 | 13.3 |
| 5 | LATERALOCCIPITAL | 0.015 | 1.274 | 0.208 | 5.1 |
|  | SUPRAMARGINAL | 0.016 | 2.848 | 0.006 | 8.3 |
|  | INFERIORPARIETAL | 0.024 | 2.858 | 0.006 | 11.7 |
|  | SUPERIORPARIETAL | 0.022 | 2.585 | 0.012 | 9.6 |
|  | PRECUNEUS | 0.019 | 2.848 | 0.006 | 20.3 |
|  | BANKSSTS | 0.019 | 2.423 | 0.019 | 9.3 |
| 6 | CUNEUS | 0.014 | 1.818 | 0.075 | 9.5 |

**Supplementary Table 5. Fitting individual variability in regional future annualised rate of tau accumulation** Parameter estimates and associated statistics for the robust regression equations using the prognostic index to predict regional future tau accumulation for individuals from ADNI 3 classified as Clinically Declining (two-tailed linear regression, uncorrected). Source data are provided as a Source Data file.

Supplementary Results

**Differences in baseline tau burden for Clinically Stable vs. Clinically Declining**

Using the model derived classification of Clinically Stable vs. Clinically Declining, we contrasted the baseline tau burden between the two groups. FTP data were realigned, and the mean of all frames was used to coregister FTP to each participant’s MRI acquired closest to the time of the FTP-PET. FTP standardised uptake value ratio (SUVR) images were normalised to inferior cerebellar grey matter ^1^. MR images were segmented and parcellated into 72 ROIs taken from the Desikan-Killiany atlas using Freesurfer (V5.3). These ROIs were then used to extract regional SUVR data from the cerebellar normalised FTP-PET images. Left and right hemisphere ROIs were averaged to generate 36 ROIs for further analysis. SUVR values in six aggregate Braak staging regions were also derived averaging uptake across individual Freesurfer region of interests (ROIs) comprising each Braak region ^2^.

Contrasting the baseline tau burden for Clinically Stable (n=59) vs. Clinically Declining (n=56) individuals showed higher baseline tau across the cortex for the Clinically Declining group (**Supplementary Figure 3a, Supplementary Table 1**). This overall pattern was consistent across all Braak regions, with a greater difference observed in earlier Braak regions (Braak I mean difference = 0.167 SUVR, t(113)=5.6,p<0.001; Braak II mean difference = 0.074 SUVR, t(113)=2.6, p=0.01; Braak III mean difference = 0.12 SUVR, t(113)=4.77 ,p<0.001; Braak IV mean difference = 0.08 SUVR, t(113)=3.81,p<0.001; Braak V mean difference = 0.07 SUVR, t(113)=3.47,p<0.001; Braak VI mean difference = 0.041 SUVR, t(113)=2.53, p=0.012) (**Supplementary Figure 3b, Supplementary Table 1**). This pattern of higher baseline tau for the Clinically Declining group was also observed in the BACS sample (Braak I mean difference=0.0445 SUVR; Braak II mean difference=0.0274 SUVR; Braak III mean difference=0.0201 SUVR; Braak IV mean difference=0.0233 SUVR; Braak V mean difference=0.0324 SUVR; Braak IV mean difference= 0.0368 SUVR) (**Supplementary Figure 4**). Note that, as there is no partial volume correction, the signal from Hippocampus (Braak II) may be unreliable.

**Comparing binary and individualised predictions with alternate modelling approaches**

**GMLVQ vs. SVM classification**

We compared the GMLVQ classification results with a linear Support Vector Machine (SVM). The SVM was run using the *fitcsvm.m* function from MATLAB statistics and machine learning toolbox. We fixed all experimental design choices when comparing the two classifiers. That is, for each resample of the data we ran the two classifiers on the same training and hold out data and calculated the class-balanced accuracy on the hold out data. To compare the performance of the two linear classifiers we performed a paired t-test on the class-balanced accuracy across resampling. Comparing the average classification performance in the ADNI2/GO training sample we observed the same model accuracy (GMLVQ: Average Accuracy 88% SVM: Average Accuracy 88%). Further, we did not observe any significant differences in classification performance across resamplings (t(798)=1.5, p=0.13). Finally, we trained each model on the full ADNI2/GO sample and generated predicted outcome labels for the ADNI 3 sample. We observe a 99.13% agreement in predicted labels for the ADNI 3 sample. Therefore, we conclude that the two low parameter linear classifiers perform comparably in the same classification task. This is likely due to several features of our classification task and classifiers used. First, both classifiers are linear and low parameter as there are only three input features used for separating two classes. As the training sample size (n=256) far exceeds the free parameters of each model, neither approach is prone to overfitting. Second, the training classes used were specifically constructed to have the best chance in finding a robust decision boundary. That is, we used multiple clinical appraisals to determine if a training target was stable cognitively normal vs. cognitively normal or MCI at baseline but received a diagnosis of Dementia. Here, we did not present the model with uncertain classes (i.e. MCI at baseline but cognitively normal or MCI at follow-up), increasing the likelihood of each classifier extracting a robust decision plane.

**GMLVQ-scalar projection vs. LTJMM prediction**

We compared the GMLVQ-scalar projection derived from baseline biomarker data with the model derived disease stage from the latent time joint mixed effects models (LTJMM) presented in Li et al. ^3^. To run the LTJMM we used the public source code for this model from <https://bitbucket.org/mdonohue/ltjmm/src/master/>.

First, we ran the LTJMM on the longitudinal FTP-PET measures in the ADNI 3 sample including as covariates APOE 4, MTL atrophy and FBP PET SUVR taken at the baseline FTP-PET scan. We modelled longitudinal FTP-PET accumulation in 7 ROIs (BANKSSTS, INFERIOR TEMPORAL, FUSIFORM, PRECUNEUS, INFERIOR PARIETAL, SUPRAMARGINAL, SUPERIOR PARIETAL). We found that the mean posterior estimates of the rate of tau accumulation for the 7 ROIs are closely associated with the observed rate of tau accumulation (mean R^2^=0.81; min R^2^=0.61; max R^2^= 0.92). Next, we extracted the latent time shift (delta) derived from the LTJMM to investigate if the model derived disease stage relates to the scalar projection. We observed a significant relationship between the LTJMM latent time shift and the scalar projection r(113)=0.42,p<0.0001. This highlights that the scalar projection derived from only baseline biomarker data relates to the LTJMM disease stage derived from both baseline and longitudinal biomarker information.

**Cognitive Classification Model**

Using the GMLVQ-Scalar projection model we trained our classifier to separate Clinically Stable (n=99) vs Clinically Declining (n=156) individuals from the ADNI2/ GO sample. We used 4 cognitive test scores as predictors to train our model: ADAS Cog, MOCA Total, MMSE Total, RAVLT Total. All cognitive assessments were taken within one year of the baseline Aβ scan. Our model achieved a cross validated class balanced classification accuracy of 86% (determined using random resampling of test data). Next, we derived the scalar projection score for the ADNI 3 sample with longitudinal tau data available. Of the 115 individuals 110 (41 MCI, 69 CN) had cognitive assessments within one year of their baseline tau scan.

The model classified 44 individuals as Clinically Declining and 66 as Clinically Stable. When comparing the agreement between the classifier and clinical diagnosis (**Supplementary Results Table 1**) we observed a fair agreement between the clinical diagnoses and the machine learning derived classification Cohen’s kappa = 0.3295, 95% CI [0.1461, 0.5129], z = 3.4388, p = 0.0006.

Next, we tested whether the classification of Clinically Declining vs Clinically Stable using the scalar projection derived from the cognitive model is sensitive to future tau accumulation. This analysis showed that the cognitive model separates individuals who will accumulate tau in the future (**Supplementary Figure 5, Supplementary Results Table 2**). Further, there was a low interclass correlation coefficient for average rate of tau accumulation between Clinically Declining and Clinically Stable individuals (r=0.07 [-0.26, 0.38], F(35,36)= 1.1492 p=0.34).

|  | Clinically Stable | Clinically Declining |
| --- | --- | --- |
| CN | 50 | 19 |
| MCI | 16 | 25 |

**Supplementary Results Table 1. Clinician vs. cognitive classifier Confusion matrix**. inter-rater reliability when diagnosing Clinically Declining based on biological predictors (i.e. scalar projection) or a clinical diagnosis based on syndromic definitions (i.e. CN or MCI).

| Braak Stage | Region | Clinically Stable (n=44) | | | Clinically Declining (n=66) | | |
| --- | --- | --- | --- | --- | --- | --- | --- |
|  |  |  | Significantly Accumulating Tau | |  | Significantly Accumulating Tau | |
|  |  | mean accumulation (SUVR/Year) | t-stat | p-val | mean accumulation (SUVR/Year) | t-stat | p-val |
| 1 | ENTORHINAL | 0.004 | 1.059 | 0.147 | 0.008 | 1.228 | 0.113 |
| 2 | HIPPOCAMPUS | 0.005 | 1.422 | 0.080 | -0.006 | -1.279 | 0.896 |
| 3 | PARAHIPPOCAMPAL | 0.003 | 0.908 | 0.184 | 0.006 | 1.180 | 0.122 |
|  | FUSIFORM | 0.004 | 1.267 | 0.105 | 0.013 | 2.627 | 0.006 |
|  | LINGUAL | 0.000 | 0.122 | 0.452 | 0.005 | 1.136 | 0.131 |
|  | AMYGDALA | 0.004 | 0.994 | 0.162 | 0.004 | 0.714 | 0.240 |
| 4 | MIDDLETEMPORAL | 0.006 | 1.566 | 0.061 | 0.019 | 3.642 | 0.000 |
|  | CAUDALANTERIORCINGULATE | 0.000 | -0.149 | 0.559 | 0.000 | -0.010 | 0.504 |
|  | ROSTRALANTERIORCINGULATE | -0.003 | -1.327 | 0.905 | -0.003 | -0.643 | 0.738 |
|  | POSTERIORCINGULATE | 0.001 | 0.408 | 0.342 | 0.007 | 2.207 | 0.016 |
|  | ISTHMUSCINGULATE | 0.002 | 0.734 | 0.233 | 0.008 | 2.399 | 0.010 |
|  | INSULA | -0.001 | -0.411 | 0.659 | 0.004 | 1.067 | 0.146 |
|  | INFERIORTEMPORAL | 0.006 | 1.563 | 0.061 | 0.018 | 3.299 | 0.001 |
|  | TEMPORALPOLE | -0.005 | -1.083 | 0.858 | -0.002 | -0.373 | 0.644 |
| 5 | SUPERIORFRONTAL | 0.002 | 0.603 | 0.274 | 0.004 | 1.144 | 0.129 |
|  | LATERALORBITOFRONTAL | 0.002 | 0.580 | 0.282 | -0.001 | -0.158 | 0.562 |
|  | MEDIALORBITOFRONTAL | -0.001 | -0.307 | 0.620 | -0.006 | -1.645 | 0.946 |
|  | FRONTALPOLE | -0.001 | -0.212 | 0.584 | 0.008 | 1.233 | 0.112 |
|  | CAUDALMIDDLEFRONTAL | 0.001 | 0.373 | 0.355 | 0.012 | 3.004 | 0.002 |
|  | ROSTRALMIDDLEFRONTAL | 0.001 | 0.326 | 0.373 | 0.005 | 1.231 | 0.113 |
|  | PARSOPERCULARIS | 0.003 | 1.040 | 0.151 | 0.005 | 1.704 | 0.048 |
|  | PARSORBITALIS | 0.003 | 0.564 | 0.287 | 0.004 | 0.816 | 0.210 |
|  | PARSTRIANGULARIS | 0.002 | 0.488 | 0.314 | 0.004 | 1.095 | 0.140 |
|  | LATERALOCCIPITAL | 0.002 | 0.441 | 0.330 | 0.025 | 3.458 | 0.001 |
|  | SUPRAMARGINAL | 0.001 | 0.462 | 0.323 | 0.011 | 2.810 | 0.004 |
|  | INFERIORPARIETAL | 0.004 | 1.063 | 0.146 | 0.020 | 3.552 | 0.000 |
|  | SUPERIORTEMPORAL | -0.001 | -0.201 | 0.580 | 0.007 | 1.924 | 0.030 |
|  | SUPERIORPARIETAL | 0.002 | 0.415 | 0.340 | 0.018 | 3.482 | 0.001 |
|  | PRECUNEUS | 0.002 | 0.768 | 0.223 | 0.008 | 2.436 | 0.010 |
|  | BANKSSTS | 0.006 | 1.740 | 0.043 | 0.011 | 2.330 | 0.012 |
|  | TRANSVERSETEMPORAL | -0.005 | -1.457 | 0.925 | -0.004 | -0.936 | 0.823 |
| 6 | PERICALCARINE | 0.001 | 0.225 | 0.411 | 0.004 | 1.029 | 0.155 |
|  | POSTCENTRAL | -0.001 | -0.445 | 0.671 | 0.007 | 1.669 | 0.051 |
|  | CUNEUS | -0.001 | -0.247 | 0.597 | 0.012 | 2.282 | 0.014 |
|  | PRECENTRAL | 0.000 | -0.048 | 0.519 | 0.006 | 1.646 | 0.054 |
|  | PARACENTRAL | 0.003 | 0.941 | 0.175 | 0.005 | 1.311 | 0.098 |
|  | Mean | 0.001 |  |  | 0.007 |  |  |

**Supplementary Results Table 2. Regional future annualised rate of tau accumulation** Measures of future regional annualised rate of tau accumulation taken from the Desikan Killiany atlas for ADNI 3 individuals within the 6 Braak stages. The mean future annualised rate of tau accumulation and test statistics describing whether a region significantly accumulates tau for individuals classified as Clinically Stable (Left block) and for individuals classified as Clinically Declining (Right block) using a cognitive classification model (one-sided t-test (right) against 0, uncorrected).

**Predicting future regional tau accumulation based on the cognitive scalar projection**

To test if the scalar projection derived from modelling the cognitive data is related to individual variability in future rate of tau accumulation we ran multiple regression models within the regions that were shown to significantly accumulate tau (**Supplementary Figure 5, Supplementary Results Table 2**). Unlike the biological scalar projection derived from modelling biological data, the cognitive scalar projection derived from modelling cognitive data did not show a significant relationship to individual variability in future rate of tau accumulation within any of the ROIs that were shown to significantly accumulate tau (**Supplementary Results Table 3**). Although the binary stratification based on cognitive data predicts future tau accumulation, the individualised score captured by the scalar projection derived from modelling cognitive data is shown to be less sensitive for fine scale stratification based on future tau accumulation. Although our cognitive model predicts whether individuals will accumulate tau in the future, it does not determine whether an individual will accumulate tau at a slow, intermediate or rapid rate.

| Braak Stage | Region | Beta Estimate (SUVR/Year) | t-stat | p-val |
| --- | --- | --- | --- | --- |
| 3 | FUSIFORM | 0.021 | 1.414 | 0.165 |
| 4 | MIDDLETEMPORAL | 0.019 | 1.005 | 0.321 |
|  | POSTERIORCINGULATE | -0.004 | -0.340 | 0.736 |
|  | ISTHMUSCINGULATE | 0.001 | 0.126 | 0.900 |
|  | INFERIORTEMPORAL | 0.018 | 0.942 | 0.351 |
| 5 | CAUDALMIDDLEFRONTAL | 0.013 | 1.152 | 0.256 |
|  | PARSOPERCULARIS | 0.002 | 0.218 | 0.829 |
|  | LATERALOCCIPITAL | 0.009 | 0.396 | 0.694 |
|  | SUPRAMARGINAL | 0.007 | 0.531 | 0.598 |
|  | INFERIORPARIETAL | 0.024 | 1.312 | 0.197 |
|  | SUPERIORTEMPORAL | -0.008 | -0.608 | 0.547 |
|  | SUPERIORPARIETAL | 0.015 | 0.923 | 0.361 |
|  | PRECUNEUS | 0.002 | 0.178 | 0.859 |
|  | BANKSSTS | 0.022 | 1.471 | 0.149 |
| 6 | CUNEUS | -0.009 | -0.513 | 0.611 |

**Supplementary Results Table 3. Fitting individual variability in regional future annualised rate of tau accumulation** Parameter estimates and associated statistics for the robust regression equations using the cognitive scalar projection to predict regional future tau accumulation for individuals from ADNI 3 classified as Clinically Declining (two-tailed linear regression, uncorrected).

**Supplementary Methods**

**Generalised Matrix Learning Vector Quantisation**

**Learning Vector Quantisation (LVQ)**

Learning Vector Quantisation (LVQ) comprises classifiers that operate in a supervised manner to iteratively modify class-specific prototypes to find boundaries of discrete classes. In particular, LVQ classifiers are defined by a set of vectors (prototypes) that represent classes within the input space. These prototypes are updated iteratively throughout the training phase, resulting in changes in class boundaries. For each training example, the closest prototype for each class is determined. These prototypes are then updated so that the closest prototype representing the same class as the input example is moved towards the input example and those representing different classes are moved further away.

Training data sample of size *n* is denoted by $\left( x_{i},y_{i} \right) \in\mathbb{R}^{m}\boldsymbol{\times}\left\{ 1\boldsymbol{,\ldots,}K \right\}\mathbf{,}i=1,2,\ldots,n$, where *m* denotes data dimensionality and *K* the number of different classes. In this instance the LVQ network comprises *L* prototypes $w_{q}\in\mathbb{R}^{m}, q=1,2,\ldots,L$defined by their location in the input space and their class label $c\left( w_{q} \right) \in\left\{ 1,\ldots,K \right\}.$

Prototypes are adapted automatically throughout training so that distances between points of class $c\in\left\{ 1,\ldots,K \right\}$ and their corresponding prototypes sharing the same label *c* is minimised, while keeping the prototypes of other classes as far as possible. Classification is then performed on test data based on a winner-takes-all process, whereby a previously unseen input vector $x_{i} \in\mathbb{R}^{m}$ is assigned the class label $c\left( w_{q} \right)$ of the closest prototype $\left( w_{q} \right)$.

**The Generalised Matrix LVQ (GMLVQ)**

The Generalised Matrix LVQ (GMLVQ) ^4^ extends the LVQ utilising a full metric-tensor for a more robust (with respect to the classification task) distance measure in the input space. To do this, the metric tensor induces feature scaling in its diagonal elements, while accounting for task conditional interactions between pairs of features (co-ordinates of the input space).

Given a positive definite matrix $\Lambda$, $\Lambda\succ0$, the generalised form of the squared distance is calculated as

$d_{\Lambda}\left( x,w \right)={(x-w)}^{T}\Lambda(x-w)$.

Positive definiteness of $\Lambda$ can be ensured by defining $\Lambda=Ω^{T}Ω$ where $Ω \in\mathbb{R}^{m \times m}$ is a full rank matrix. Note that only relative distances of input points to the prototypes are important. Hence, the metric tensor can be multiplied by any positive real number without effecting the classifier performance in any way. To account for this inherently ill-posed nature of the model fitting and hence to ensure the stability of the algorithm, $\Lambda$ must be normalised following each learning step e.g. by making sure that $\sum_{i} \Lambda_{i,i}=1$, fixing the trace throughout learning.

Using the steepest descent method, the cost function to be minimised through online learning is

$$f_{\mathrm{GMLVQ}}=\sum_{i=1}^{n} \varphi\left( \mu_{\Lambda}(x_{i}) \right)$$

where

$$\mu_{\Lambda}\left( x_{i} \right)=\frac{d_{\Lambda}\left( x_{i},w^{+} \right)-d_{\Lambda}\left( x_{i},w^{-} \right)}{d_{\Lambda}\left( x_{i},w^{+} \right)+d_{\Lambda}\left( x_{i},w^{-} \right)}$$

In this instance $\varphi$ is a monotonic identity function$\varphi\left( \mathcal{l} \right)\mathcal{=l}$, $d_{\Lambda}\left( x_{i},w^{+} \right)$ is the distance between the sample vector $x_{i}$ from the closest prototype with the same class label ${c(w)}^{+}={c(x}_{i})$ and $d_{\Lambda}\left( x_{i},w^{-} \right)$is the distance from $x_{i}$ to the closest prototype of a different class. We assessed model performance by classification accuracy, true positive rate, true negative rate and averaged error (i.e. average error of false positive and false negative).

**Interrogating the Metric Tensor Matrix:**

The metric tensor provides a way to determine how predictive different data is in the classification task. Below are toy examples highlighting how the elements of the metric tensor transform the feature space by weighting univariate and pairwise features. The examples show a hypothetical binary classification task. The data and metric tensor are shown in the original space and in the learnt space (i.e. after feature scaling by the metric tensor). The metric tensor is learnt to ensure that all points have a neighbour of the same class, that is, the shortest pairwise distance for all points is with a point from the same class.

Here, we provide 3 examples of how the elements of the metric tensor scales the data in the input space and the interpretation of the metric tensor.

**Panel a:** All examples show the initialised metric tensor on the left panel, where the diagonal values are both equal to 0.5 and the off diagonals are equal to 0. The panel on the right gives an example of the data distribution in the original space, where red circles are training data of class 1 and blue dots are of class 2. The solid black line represents the X and Y axis in the original space.

**Panel b:** All examples show the learnt metric tensor following the GMLVQ process on the left panel, note the diagonal terms sum to 1 and the off diagonals can be either negative of positive. The right panel shows the transformed space when scaled by the learnt metric tensor: the red circles and the blue dots are the same individuals from **Panel a** in the transformed space following the GMLVQ process and the solid black lines show the transformation of the X and Y axis into the learnt space.

**Example 2.1: Univariate weights**

**
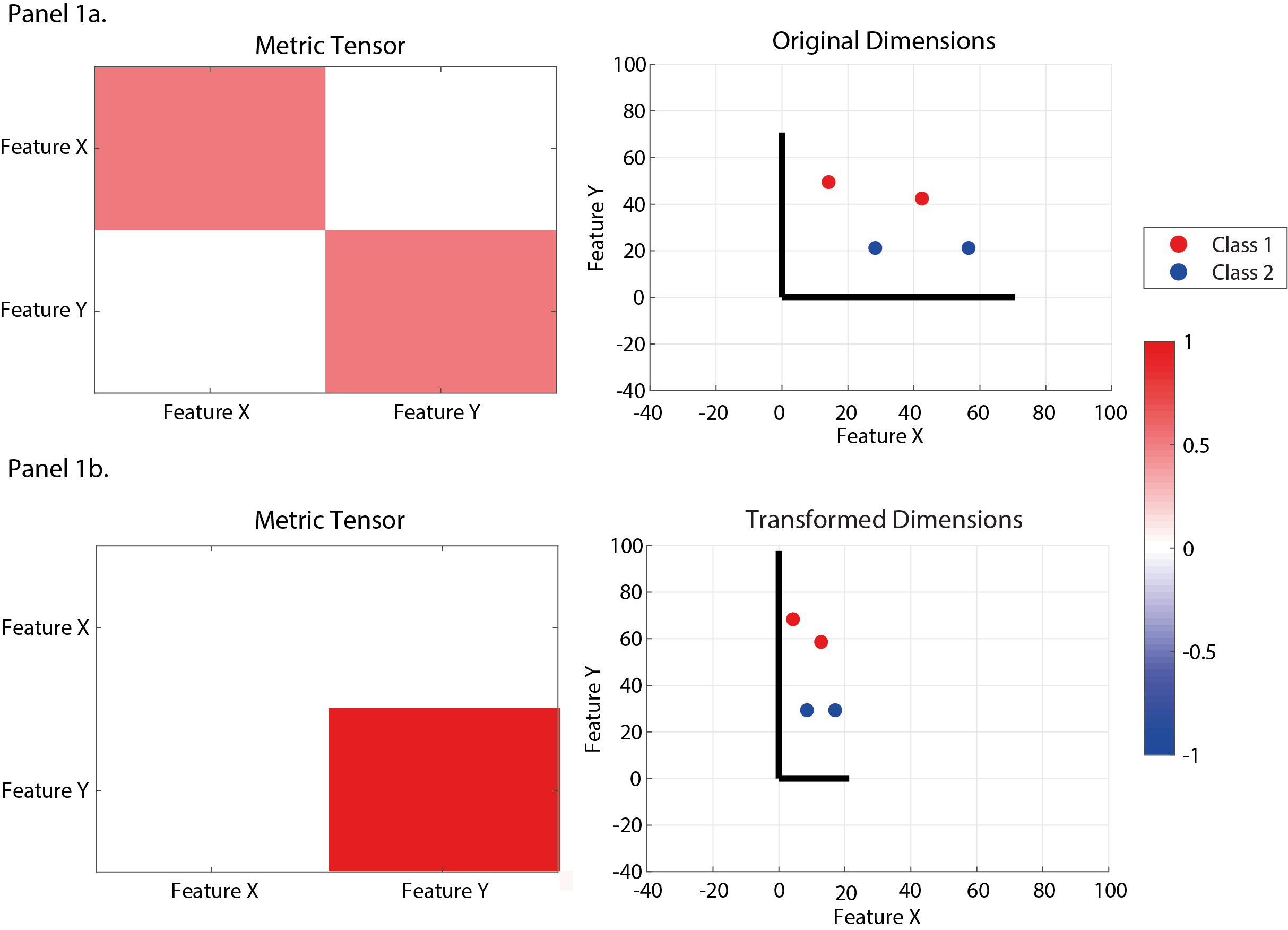
**

**Example 2.1** shows that for all data have a neighbour of the same class the diagonals need to be scaled so that the term for Feature Y has to be increased relative to Feature X The conclusion that is drawn from the metric tensor is that Feature Y is the most relevant feature.

**Example 2.2 Multivariate weights (positive)**

**
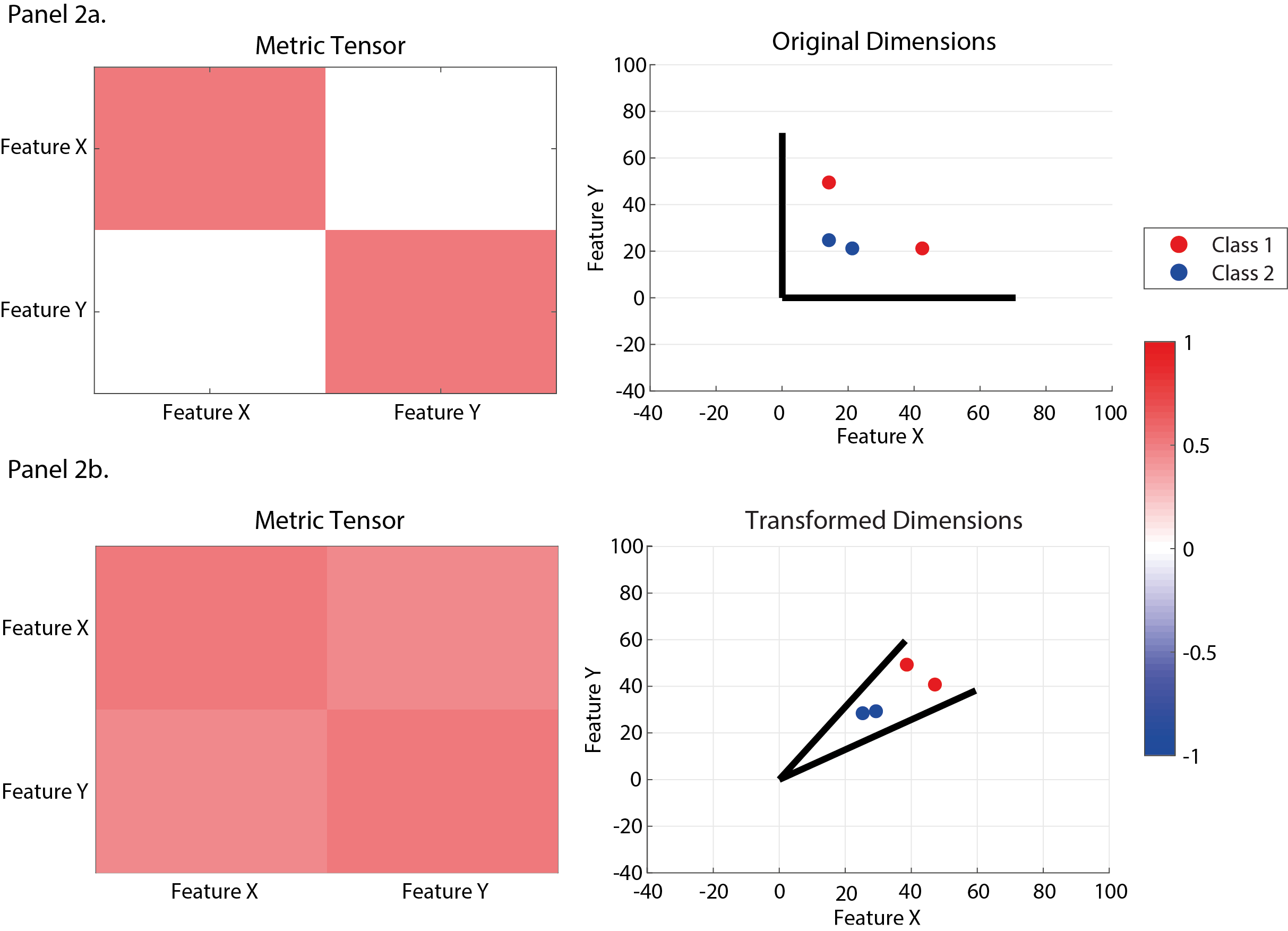
**

**Example 2.2**, the interaction between Feature X and Feature Y is highly relevant to group training samples of the same class (Note that the off-diagonal terms are positive). This interaction term collapses the angle between the two dimensions causing data of the same class to group together.

**Example 2.3 Multivariate weights (negative)**

**
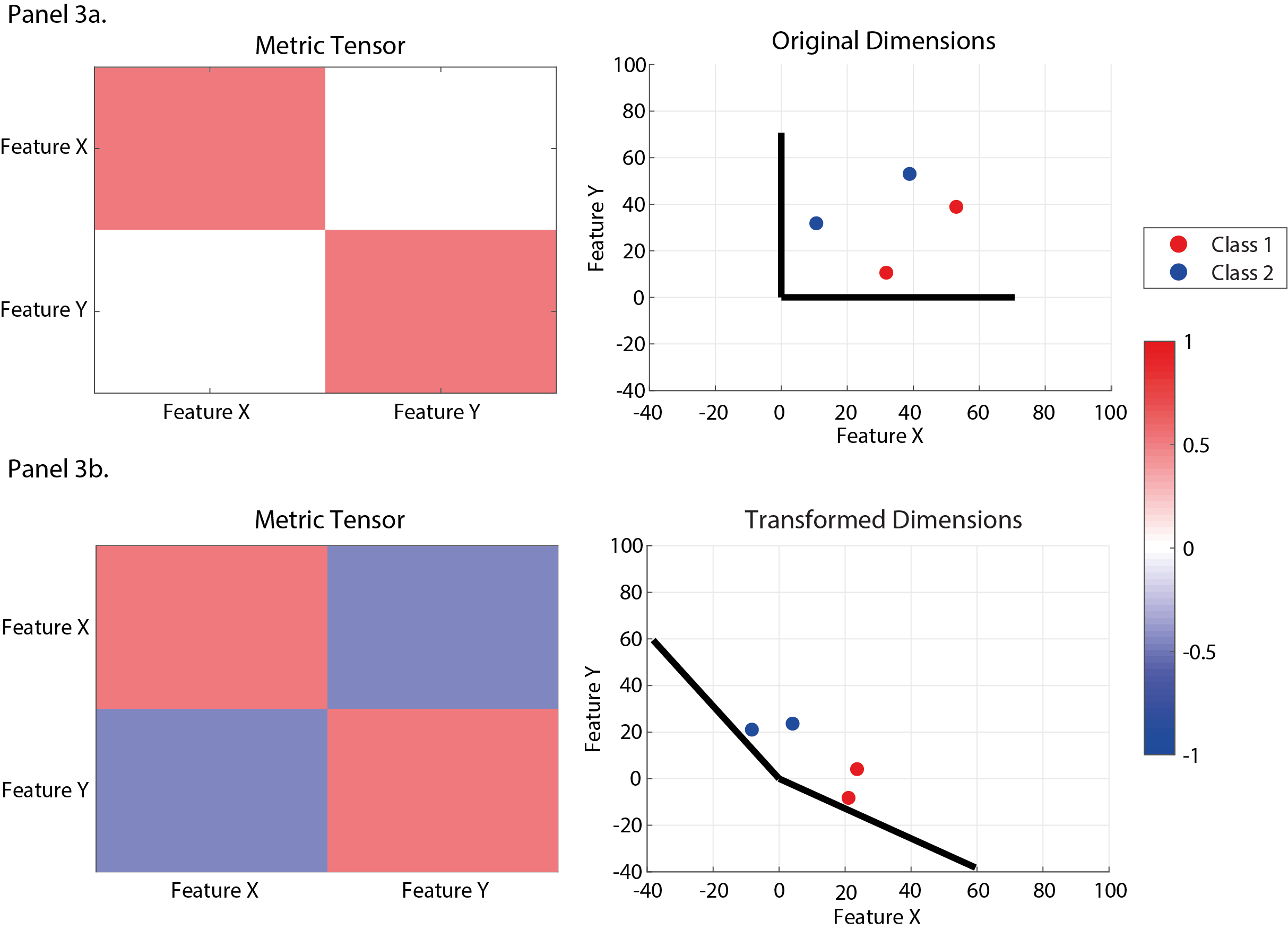
**

**Example 2.3** shows that for all data to have a neighbour of the same class the interaction between Feature X and Feature Y is relevant; however, as this term is negative it increases the angle between the two dimensions. Thus, we conclude that these two features separate points from different classes.

To interpret these examples mathematically, note that the squared distance can be written as

$d_{\Lambda}\left( x,w \right)=\left( x-w \right)^{T}\Lambda\left( x-w \right)= \left( X-W \right)^{T}\left( X-W \right)= d_{2}\left( X,W \right)$,

where $d_{2}\left( .,. \right)$ denotes the squared Euclidean distance and ${X=\Lambda}^{1/2}x$, ${W=\Lambda}^{1/2}w$. Hence, the original vectors (including the unit directional vectors of the standard axis) are transformed by the linear operator equal to the square root of the symmetric positive-definite metric tensor $\Lambda$ into a space where the new distance (defined by $\Lambda$) can be re-interpreted as the usual Euclidean distance.

**GMLVQ – Scalar Projection Distance to Clinically Stable Prototype**

The GMLVQ- Scalar Projection method extends the GMLVQ framework to extract specific distance information from the sample vector $x_{i}$ and the learnt prototypes$w_{(Clinically Stable,Clinically Declining)}$.

Specifically, we determine the distance in the learnt space (i.e. after applying the learnt metric tensor) between an individual with sample vector $x_{i}$and the learnt prototype $w_{Clinically Stable}$ along the vector separating $w_{Clinically Stable}$ and $w_{Clinically Declining}$

Following the learning process in GMLVQ we transformed the sample vector $x_{i}$ and prototypes $w_{(Clinically Stable,Clinically Declining)}$into the learnt space via the metric tensor$\Lambda$.

$$X_{i}=\Lambda^{1/2} x_{i}$$

$$W_{(Clinically Stable,Clinically Declining)}=\Lambda^{1/2}w_{(Clinically Stable,Clinically Declining)}$$

As the metric tensor $\Lambda$ is learnt in the non-Euclidean space:$d_{\Lambda}\left( x,w \right)={(x-w)}^{T}\Lambda(x-w)$, we applied the square root of this tensor to re-represent the data so that the squared norm of a vector in the non-Euclidian space is equal to the squared Euclidean norm of the transformed space.

We centred the coordinate system on $W_{(Clinically Stable)}$ and calculated the orthogonal projection of each vector $X_{i}$ onto the vector $W_{\left( Clinically Declining \right)}$, in this co-ordinate system.

$$Projection=\frac{X_{i}W_{Clinically Stable}.W_{Clinically Declining}W_{Clinically Stable}}{\left| W_{Clinically Declining}W_{Clinically Stable} \right|}$$

To normalise the projections with respect to the position of the prototype $W_{(Clinically Stable)}$, we divided the projection by the norm of $W_{Clinically Stable}W_{Clinically Declining}$:

$$Scalar Projection=\frac{X_{i}W_{Clinically Stable}.W_{Clinically Declining}W_{Clinically Stable}}{\left| W_{Clinically Decliing}W_{Clinically Stable} \right|^{2}}$$

The resultant value indicates the separation of a test point from prototype $W_{Clinically Stable}$ along the direction of $W_{Clinically Decliing}W_{Clinically Stable}$. To determine the relative separation from the stable prototype, we normalised the projection by the distance between each prototype $W_{Clinically Decliing},W_{Clinically Stable}$, as indicated by squaring the norm of vector $W_{Clinically Decliing}W_{Clinically Stable}$. A large positive projection indicates a large separation from $W_{Clinically Stable}$ in direction $W_{Clinically Decliing}W_{Clinically Stable}$ and a large negative projection indicates a large separation from $W_{Clinically Stable}$in the opposite direction i.e. $W_{Clinically Stable}W_{Clinically Decliing}$. In particular, a value of 1 indicates that a sample is incident to prototype $W_{Clinically Decliing},$whereas a value of 0 indicates that a sample is incident to prototype $W_{Clinically Stable}$. A value of 0.5 is the decision boundary separating the two classes within the binary classification framework. That is, the scalar projection has a large positive value for Clinically Declining individuals and zero or negative value for Clinically Stable individuals.

#### Graphical Interpretation

#### Deriving the GMLVQ – Scalar Projection: Prototype Vector

The figure below uses the data from Example 2 above to graphically derive the Prototype Vector ($W_{q}W_{j})$ that is generated following the GMLVQ learning process. Note: Here we return to general notation, where class 1 is *Clinically Declining* and class 2 is *Clinically Stable* in the description above.

**Figure 2.3** **Prototype vector**


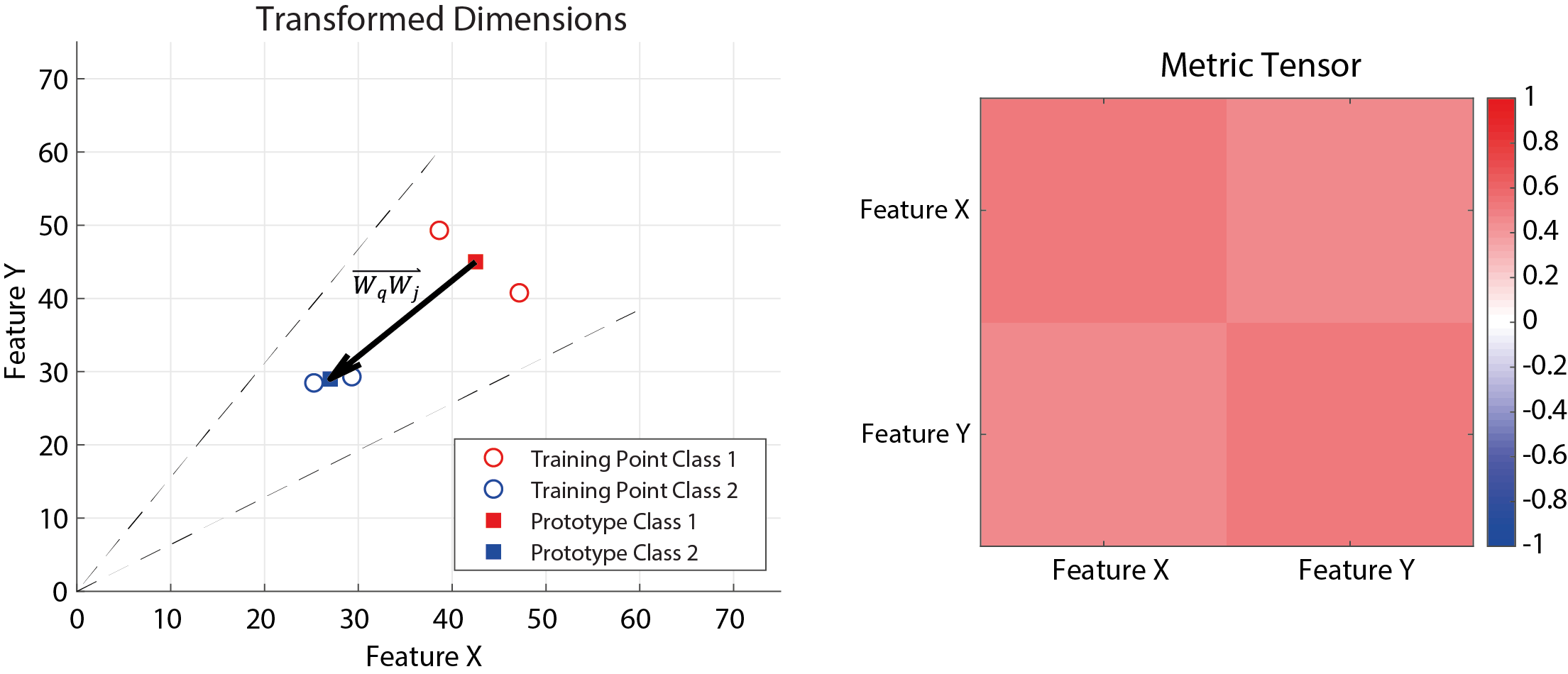


**Figure 2.3** The left panel shows the vector $W_{q}W_{j}$ represented by the black arrow connecting prototypes for class 1 and class 2. The dashed lines show the transformed axis from the original space. The right panel is the learnt metric tensor used to transform the data from the original space into the learnt space.

#### Deriving the GMLVQ – Scalar Projection: Test Projections

Here, we use the data and learnt metric tensor from **Example 2.2** in **Interrogating the Metric Tensor Matrix** to show three scenarios where values for the scalar projection are calculated and interpreted in a general sense.

The left panel shows a possible test point in the original data space of **Example 2.2**. The red dots are the training sample for class 1, the blue dots are the training sample for class 2 and the black X is the test point in the original space.

The right panel shows the graphical derivation of the scalar projection in the learnt space for the test data point X. Here, the red dots are the training sample for class 1 and the blue dots are the training sample for class 2, the black X is the test point in the learnt space (i.e. after the GMLVQ learning process) and the dashed black lines show the transformation of the X and Y axis into the learnt space.

The red square is the prototype for class 1 in the learnt space, the blue square is the prototype for class 2 in the learnt space. The vector $W_{q}W_{j}$ connects the prototypes for class 1 and class 2, and vector $X_{i}W_{j}$ connects the test point in the learnt space with the prototype for class 2. The dashed blue line is the orthogonal projection from the test point in the learnt space to vector $W_{q}W_{j}$. The solid blue line represents the scalar projection from the test point in the learnt space to the class 2 prototype along the vector $W_{q}W_{j}$.

**Example 2.4 Between two classes**

**
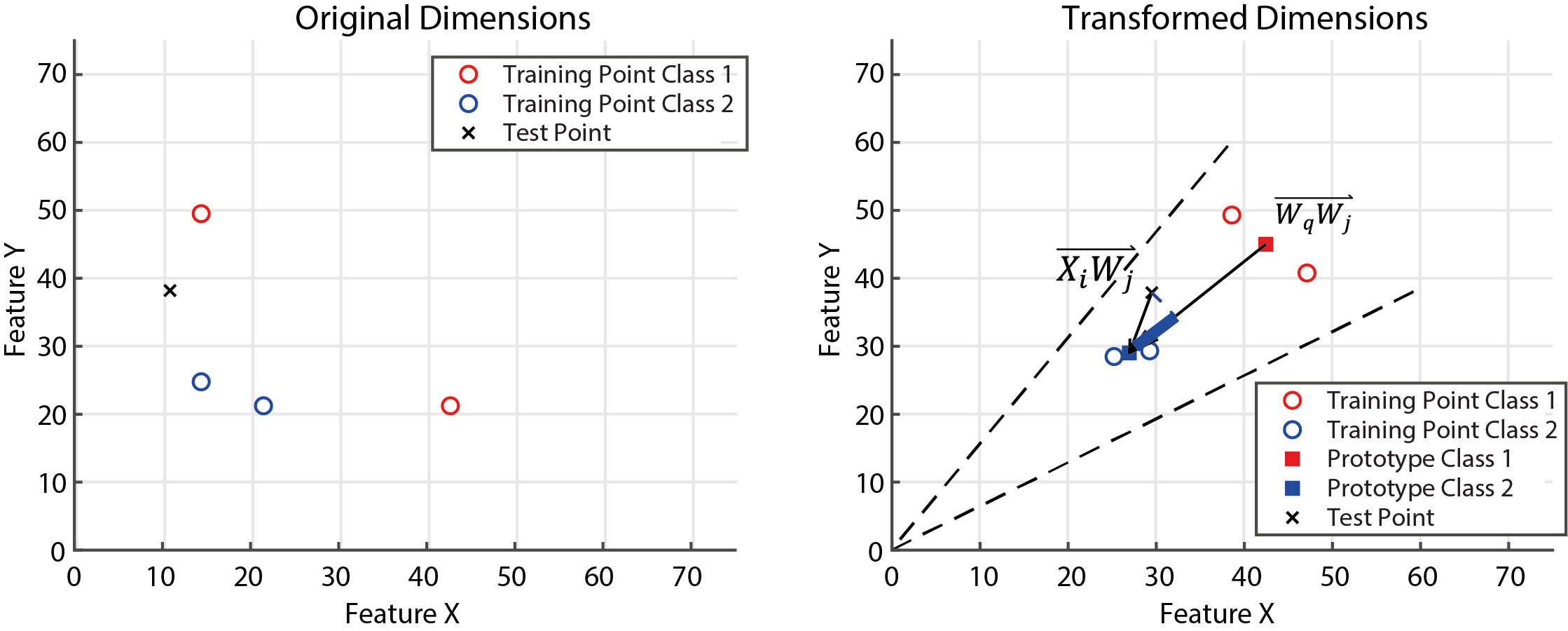
**

**Example 2.4** shows an example of a scalar projection that has a length between 0 and 1. Within the binary classification framework a decision boundary exists at 0.5, where a value below this will cause the point to be labelled as class 2 and a value beyond this will cause the point to be labelled class 1. This is interpreted as an individual that is between the two classes.

**Example 2.5 Positive value greater than one**

**
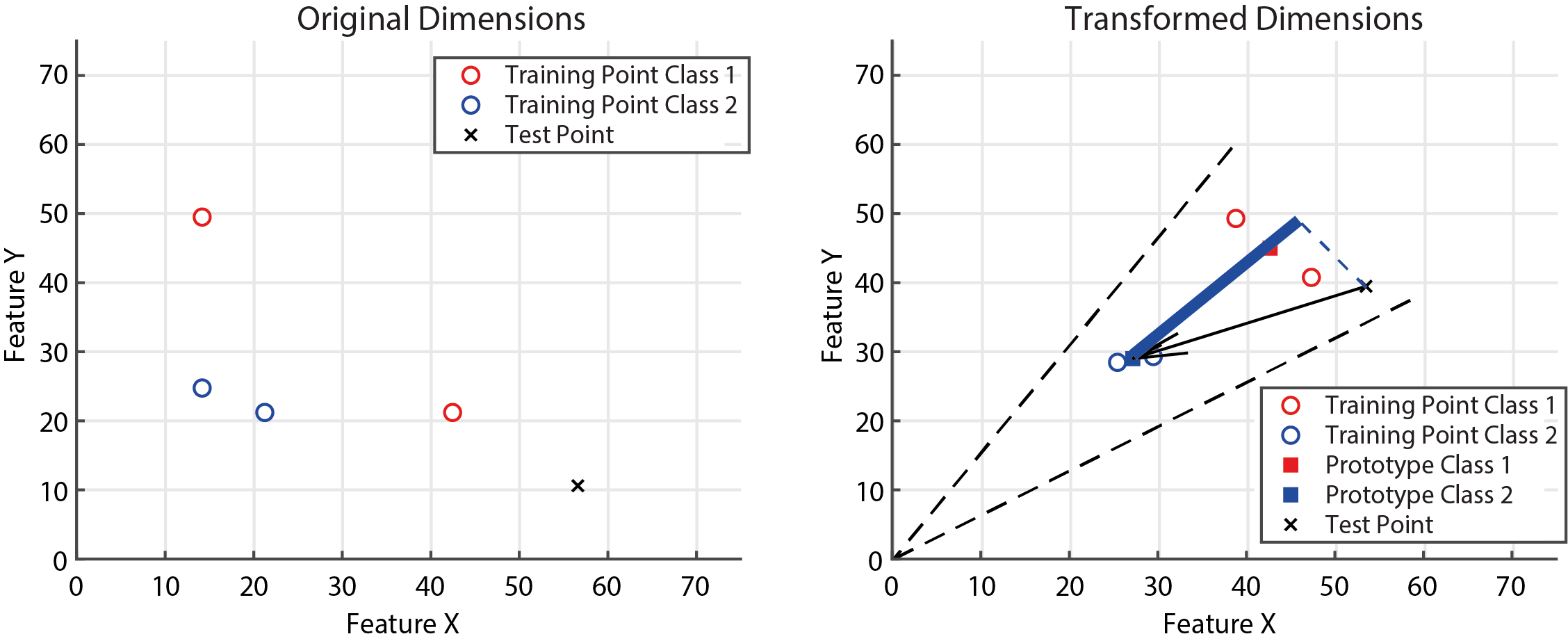
**

**Example 2.5** is given where the length of the scalar projection is larger than 1, in this example the individual is further away from class 2 prototype along the prototype vector than the class 1 prototype. This is interpreted as being further away from the class 2 prototype than the majority of class 1 individuals.

**Example 2.6 Negative value**

**
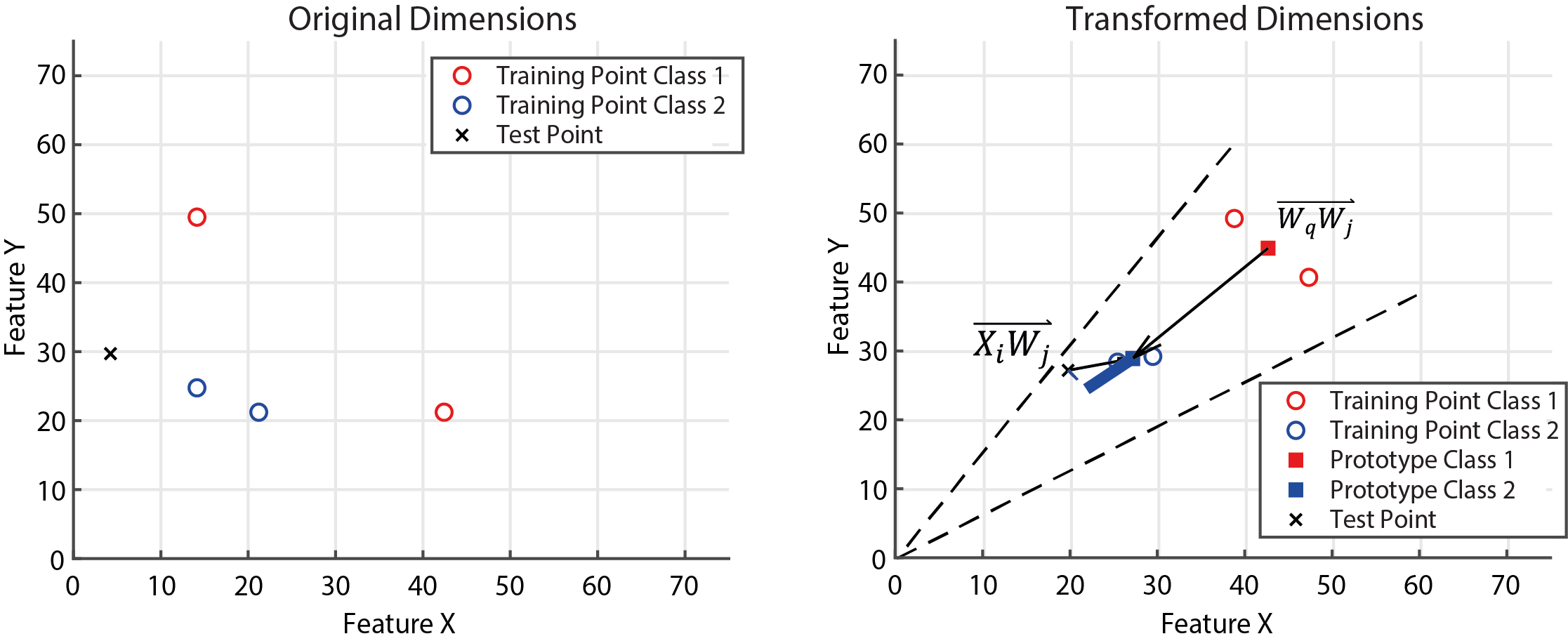
**

**Example 2.6** shows an example where the length of the scalar projection is negative. In this example the individual is further away from the class 1 prototype than the class 2 prototype along the prototype vector. This is interpreted as being further away from the class 1 prototype than the majority of class 2 individuals.

**GMLVQ – Scalar Projection: Summary/Conclusion**

The GMLVQ-scalar projection approach is a method for deriving a continuous predictive variable from the GMLVQ binary classification framework. Our methodology defines a novel way of learning a continuous prognostic metric while training using ‘noisy’ diagnostic labels. The development of this approach was motivated to capitalise on broad class labels to train a model and derive an individual continuous score. By building a model to separate two continuous target values into two general categories (i.e. Clinically Stable or Clinically Declining), we have forced the model to concentrate on the main structures in the data that lie between distinguishing the higher and lower target values. As our model has relatively - compared to deep learning approaches- few degrees of freedom (DOF), the model is unlikely to overfit the training data.

Where DOF for the model is DOF for prototypes + DOF for metric tensor.

DOF for the prototypes = D*C*P, and, metric tensor = D^2^- (D^2^-D)/2

D = number of original input features (input dimensions)

C = number of classes

P = number of prototypes per class

The GMLVQ-scalar projection approach addresses three inherent issues with prognostic models in AD research. 1.) The GMLVQ-scalar projection approach is able to account for target uncertainty. This is achieved by having a model that learns a low-parameter task-dependent scaling matrix (metric tensor), and only two locations in hyperdimensional space (prototypes). These univariate (diagonal) and multivariate (off diagonal) relationships are learnt to separate the two classes (Clinically Stable vs Clinically Declining) as best possible from a global perspective (vs local metric tensors) without over constraining the predictor data. Similarly, by defining only one position in this learnt space that best determines if a person is Clinically Stable or Clinically Declining, the model must ignore subtle differences for any given target, learning a broad location that best describes Clinically Stable / Clinically Declining populations. By not over constraining the data, this type of model will not be sensitive enough to overfit based on subtle difference in diagnostic criteria or by patients on the class boarder. 2.) The GMLVQ-scalar projection approach is able to derive a continuous metric of how far an individual is from the Clinically Stable prototype along the dimension that best separates Clinically Stable and Clinically Declining training samples. This allows the model to learn implicitly a continuous prognostic trajectory for an individual that may be predictive of underlying pathophysiological change that leads to a deteriorating condition (i.e. Clinically Declining pathophysiology). 3.) The GMLVQ-scalar projection approach is perfectly suited to harmonise data that was collected using diagnostic criteria based on syndromic definitions with inherently poor sensitivity and specificity to extract a highly sensitive continuous prognostic index.

**References**

1. Baker, S. L., Maass, A. & Jagust, W. J. Considerations and code for partial volume correcting [18F]-AV-1451 tau PET data. *Data Br.* **15**, 648–657 (2017).

2. Maass, A. *et al.* Comparison of multiple tau-PET measures as biomarkers in aging and Alzheimer’s disease. *Neuroimage* **157**, 448–463 (2017).

3. Li, D., Iddi, S., Thompson, W. K. & Donohue, M. C. Bayesian latent time joint mixed effect models for multicohort longitudinal data. *Stat. Methods Med. Res.* **28**, 835–845 (2019).

4. Schneider, P., Biehl, M. & Hammer, B. Adaptive Relevance Matrices in Learning Vector Quantization. *Neural Comput.* **21**, 3532–3561 (2009).
