# Supplementary material for: A robust and interpretable machine learning approach using multimodal biological data to predict future pathological tau accumulation
Source: Nat Commun. 2022 Apr 7;13:1887. doi: 10.1038/s41467-022-28795-7 (PMC8989879; doi:10.1038/s41467-022-28795-7)
Supplement: Supplementary file 3 — Description to Supplementary Information [file 41467_2022_28795_MOESM3_ESM.pdf]

Supplementary Figure 1: Distributions of biological predictors for Clinically Stable, Clinically Declining and Alzheimer's Clinical Syndrome groups.

Supplementary Figure 2. Effect of MRI field strength on scalar projection (BACS)

Supplementary Figure 3. ADNI 3 difference in baseline tau burden Clinically Declining vs. Clinically Stable

Supplementary Figure 4. BACS difference in baseline tau burden Clinically Declining vs. Clinically Stable

Supplementary Figure 5. Regional future annualised rate of tau accumulation CN vs. MCI

Supplementary Figure 6. Regional future annualised rate of tau accumulation across the 36 Desikan Killiany ROIs. Classification of Clinically Declining vs Clinically Stable individuals using cognitive data from the ADNI 3 sample

Supplementary Figure 7. Relationship of medial temporal lobe (MTL) grey matter density score and total intracranial volume (TIV).

Supplementary Table 1. ADNI 3 difference in baseline tau burden Clinically Declining vs. Clinically Stable.

Supplementary Table 2. Regional future annualised rate of tau accumulation Clinically Declining vs. Clinically Stable

Supplementary Table 3. Regional future annualised rate of tau accumulation CN vs. MCI

Supplementary Table 4. Regional Future Annualised Rate of Tau Accumulation Clinically Declining vs. β-amyloid Positive

Supplementary Table 5. Fitting individual variability in regional future annualised rate of tau accumulation

Supplementary Results.

Supplementary Methods.
