# Supplementary material for: A robust and interpretable machine learning approach using multimodal biological data to predict future pathological tau accumulation
Source: Nat Commun. 2022 Apr 7;13:1887. doi: 10.1038/s41467-022-28795-7 (PMC8989879; doi:10.1038/s41467-022-28795-7)
Supplement: Supplementary file 5 — Reporting Summary [file 41467_2022_28795_MOESM5_ESM.pdf]

nature research

Corresponding author(s): Zoe Kourtzi

Last updated by author(s): Jan 19, 2022

Reporting Summary

Nature Research wishes to improve the reproducibility of the work that we publish. This form provides structure for consistency and transparency in reporting. For further information on Nature Research policies, see our Editorial Policies and the Editorial Policy Checklist.

Statistics

For all statistical analyses, confirm that the following items are present in the figure legend, table legend, main text, or Methods section.

n/a	Confirmed	
☐	☒	The exact sample size (*n*) for each experimental group/condition, given as a discrete number and unit of measurement
☐	☒	A statement on whether measurements were taken from distinct samples or whether the same sample was measured repeatedly
☐	☒	The statistical test(s) used AND whether they are one- or two-sided<br>*Only common tests should be described solely by name; describe more complex techniques in the Methods section.*
☐	☒	A description of all covariates tested
☐	☒	A description of any assumptions or corrections, such as tests of normality and adjustment for multiple comparisons
☐	☒	A full description of the statistical parameters including central tendency (e.g. means) or other basic estimates (e.g. regression coefficient) AND variation (e.g. standard deviation) or associated estimates of uncertainty (e.g. confidence intervals)
☐	☒	For null hypothesis testing, the test statistic (e.g. *F*, *t*, *r*) with confidence intervals, effect sizes, degrees of freedom and *P* value noted<br>*Give P values as exact values whenever suitable.*
☒	☐	For Bayesian analysis, information on the choice of priors and Markov chain Monte Carlo settings
☒	☐	For hierarchical and complex designs, identification of the appropriate level for tests and full reporting of outcomes
☐	☒	Estimates of effect sizes (e.g. Cohen's *d*, Pearson's *r*), indicating how they were calculated

Our web collection on statistics for biologists contains articles on many of the points above.

Software and code

Policy information about availability of computer code

Data collection	no software was used
Data analysis	Machine learning implementation and analysis: MATLAB 2019a, MATLAB statistics and machine learning toolbox.<br>Custom code used in this work is available via the same link to the University of Cambridge online data repository XX.<br>Structural MRI: Statistical Parametric Mapping 12 , DARTEL<br>PET: FreeSurfer v5.3<br>Robust Correlation: Robust-Correlations (CPernet) https://github.com/CPernet/Robust-Correlations<br>LTJMM: https://bitbucket.org/mdonohue/ltjmm/src/master/

For manuscripts utilizing custom algorithms or software that are central to the research but not yet described in published literature, software must be made available to editors and reviewers. We strongly encourage code deposition in a community repository (e.g. GitHub). See the Nature Research guidelines for submitting code & software for further information.

Data

Policy information about availability of data

All manuscripts must include a data availability statement. This statement should provide the following information, where applicable:

- Accession codes, unique identifiers, or web links for publicly available datasets
- A list of figures that have associated raw data
- A description of any restrictions on data availability

The summary data generated in this study have been deposited in the University of Cambridge online data repository under accession code XX. ADNI data is accessible via adni.loni.usc.edu. Additional BACS data is available on request. Source data are provided with this paper.

Field-specific reporting

Please select the one below that is the best fit for your research. If you are not sure, read the appropriate sections before making your selection.

☒ Life sciences ☐ Behavioural & social sciences ☐ Ecological, evolutionary & environmental sciences

For a reference copy of the document with all sections, see nature.com/documents/nr-reporting-summary-flat.pdf

Life sciences study design

All studies must disclose on these points even when the disclosure is negative.

Sample size	All possible ADNI and BACS data that were available were used in the preparation of this manuscript. No initial power calculations were performed.
Data exclusions	10 ADNI 3 subjects were excluded as they did not have either a FBP PET scan or a MRI scan within 365 days of the FTP PET scan.
Replication	To validate the ability to predict individualised trajectories of future tau accumulation we use a replication sample of 56 community dwelling cognitively normal participants from BACS. Replication experiments were run only when all preliminary analysis passed acceptance criteria as described in the methods. All replication experiments had the same trends with prediction variances similar to the ones presented in the manuscript.
Randomization	There was no allocation of participants into experimental groups. Demographic recruitment criteria were similar between the ADNI and BACS studies. Post hoc analyses showed a negligible effect of demographic covariates (Age, Sex, Education).
Blinding	Group allocation was determined by cohort, ADNI2/GO: Training, ADNI 3 & BACS: Validation. Data collection was performed by independent groups who were not involved in the analysis or design of the project. As group assignment was determined by cohort, analysis was not blinded to group allocation. Analysis was blinded to group demographics (Age, Sex, Education).

Reporting for specific materials, systems and methods

We require information from authors about some types of materials, experimental systems and methods used in many studies. Here, indicate whether each material, system or method listed is relevant to your study. If you are not sure if a list item applies to your research, read the appropriate section before selecting a response.

Materials & experimental systems

n/a	Involved in the study	
☒	☐	Antibodies
☒	☐	Eukaryotic cell lines
☒	☐	Palaeontology and archaeology
☒	☐	Animals and other organisms
☐	☒	Human research participants
☒	☐	Clinical data
☒	☐	Dual use research of concern

Methods

n/a	Involved in the study	
☒	☐	ChIP-seq
☒	☐	Flow cytometry
☐	☒	MRI-based neuroimaging

Human research participants

Policy information about studies involving human research participants

Population characteristics	Data from 437 individuals from ADNI 2/GO were used to train the machine learning model. Individuals were placed into three categories based on their baseline and longitudinal syndromic labels from clinical diagnosis independent of their baseline biomarker status, with baseline defined as the evaluation closest to the first florbetapir (FBP) PET scan acquired in ADNI.<br>Alzheimer's Clinical Syndrome (n=181, 158 Aß+ at baseline, APOE 4(+/-)=119/62, Age mean=73.7+-std=6.3 years, Education mean=16.7+-std=2.7 years, Sex (M/F)=107/74): individuals have a stable diagnosis of dementia (in ADNI this corresponds to AD); Clinically Stable (n=100, 18 Aß+ at baseline, APOE 4(+/-)=21/79, Age mean=73.7+-std=6.3 years, Education mean=16.7+-std=2.7 years, Sex (M/F)=51/49): individuals have a baseline diagnosis of cognitively normal and retain this diagnosis at follow up for 4 or more years (mean=5.7+-std=1.1 years); Clinically Declining (n=156, 130 Aß+ at baseline, APOE 4(+/-)=95/61, Age mean=74.9+-std=7 years, Education mean=15.9+-std=2.7 years, Sex (M/F)=88/68): individuals have a baseline diagnosis (at date of FBP scan) of either cognitively normal (n=17) or MCI (n=139) but received a diagnosis of demented in future clinical evaluation (i.e. progressed to dementia (n=75), or had been diagnosed as demented in a clinical evaluation prior to baseline (i.e. reverted (n=81).<br>Data from 115 individuals from ADNI 3 were used to test the relationship between the prognostic index and regional future tau accumulation. These individuals were either cognitively normal (n=72) or MCI (n=43) (61 Aß+ at baseline, APOE 4(+/-)=51/64, Age mean=73.7+-std=6.9 years, Education mean=16.6+-std=2.3 years, Sex (M/F)=58/57) at baseline, defined as the diagnosis closest to the first flortaucipir (FTP) PET scan acquired in ADNI 3, and have at least one follow-up FTP PET scan.

Data from 56 community dwelling individuals from BACS were used to test the accuracy of predictions of regional future tau accumulation. These individuals were cognitively normal (n=56, 30 Aß+ at baseline, APOE 4(+/-)=17/39, Age mean=77.2+-std=5.2 years, Education mean=16.7+-std=1.8 years, Sex (M/F)=22/34) at baseline (defined as the diagnosis closest to the first FTP PET scan acquired in BACS) and have at least one follow-up FTP PET scan.

Recruitment	All ADNI subjects were recruited within the Alzheimer's Disease Neuroimaging Initiative (ADNI, see http://adni.loni.usc.edu/) the investigators of the study were not involved in ADNI subject recruitment. Recruitment for BACS occurred at Lawrence Berkeley National Laboratory (LBNL) and the University of California, Berkeley. Primary analysis was performed by investigators not involved in the BACS recruitment process.
Ethics oversight	For ADNI, ethical approval was obtained by the ADNI investigators, all participants provided written informed consent. The institutional review board (IRB) at Lawrence Berkeley National Laboratory (LBNL) and the University of California, Berkeley approved the BACS project, and written, informed consent was obtained from all BACS participants. Primary analysis was performed by investigators not involved in the BACS recruitment process.

Note that full information on the approval of the study protocol must also be provided in the manuscript.

Magnetic resonance imaging

Experimental design

Design type	Structural MRI
Design specifications	n/a
Behavioral performance measures	n/a

Acquisition

Imaging type(s)	Structural MRI
Field strength	1.5T and 3T
Sequence & imaging parameters	Structural MRIs for the ADNI samples were acquired at ADNI-GO, ADNI-2 and ADNI-3 sites equipped with 3 T MRI scanners using a 3D MP-RAGE or IR-SPGR T1-weighted sequences, as described online (http://adni.loni.usc.edu/methods/documents/mri-protocols). Structural MRIs for the BACS sample were collected on either a 1.5T MRI scanner at Lawrence Berkeley National Laboratory (LBNL) or a 3T MRI scanner at UC Berkeley using 3D MP-RAGE T1- weighted sequences. All ADNI and BACS scans were acquired with voxel sizes of approximately 1mm X 1mm X 1mm.
Area of acquisition	Whole Brain

Diffusion MRI ☐ Used ☐ Not used

Preprocessing

Preprocessing software	SPM 12
Normalization	Structural scans were segmented into grey matter, white matter and Cerebrospinal Fluid (CSF). The DARTEL toolbox 59 was then used to generate a study specific template to which all scans were normalised. Following this, individual grey matter segmentation volumes were normalised to MNI space without modulation.
Normalization template	MNI
Noise and artifact removal	n/a
Volume censoring	n/a

Statistical modeling & inference

Model type and settings	Feature extraction
Effect(s) tested	n/a

Specify type of analysis: ☒ Whole brain ☐ ROI-based ☐ Both

Statistic type for inference (See Eklund et al. 2016)	n/a
Correction	n/a

Models & analysis

n/a	Involved in the study
☒	☐ Functional and/or effective connectivity
☒	☐ Graph analysis
☐	☒ Multivariate modeling or predictive analysis

Multivariate modeling and predictive analysis

To generate a single index of medial temporal grey matter density we used a voxel weights matrix that was previously derived to generate an interpretable and interoperable disease-specific biomarker 19. In brief, a feature generation methodology (partial least squares regression with recursive feature elimination (PLSr-RFE)) was used to apply a decomposition on a set of predictors (T1-weighted MRI voxels) to create orthogonal latent variables that show the maximum covariance with the response variable (memory score). Further, we performed recursive feature elimination by iteratively removing predictors (voxels) that have weak predictive value. The PLSr-RFE procedure results in a voxel weights matrix that is used to calculate a single score of AD related medial temporal atrophy. This index of medial temporal grey matter density has been shown to predict memory deficits, relate to individual tau burden and discriminates stable MCI and progressive MCI individuals 19. To generate an individual's score of medial temporal grey matter density we performed a matrix multiplication of the previously derived voxel weights matrix and each subject's pre-processed T1 weighted MRI scans.
